# Prestige, Pride, and Belief: A Hypothesized Neural Integration Framework for Status, Identity, and Modern Polarization

**DOI:** 10.3390/brainsci16080847

**Published:** 2026-08-10

**Authors:** Sarfaraz K. Niazi

**Affiliations:** College of Pharmacy and Pharmaceutical Sciences, Washington State University, Spokane, WA 99202, USA; s.niazi@wsu.edu

**Keywords:** prestige neurobiology, dopaminergic reward, authentic and hubristic pride, sacred values, identity fusion, social neuroscience, digital polarization, intergroup contact

## Abstract

**Highlights:**

**What are the main findings?**

- Prestige, pride, ownership, sacred values, and digital status seeking can be organized within a single provisional framework, the Hypothesized Neural Integration Model of Prestige and Pride, which groups five categories of functionally pluripotent processing engaging nodes within overlapping reward, default-mode, salience, and neuroendocrine systems rather than five dissociable regional mechanisms.
- Direct neuroimaging evidence is strongest for social rank and material ownership, moderate for sacred-value processing and adolescent peer feedback and limited or extrapolated for prestige in the strict sense of freely conferred deference, for digital prestige loops, and for ideological hostility. Identity fusion is supported far more by behavioral than by neural evidence.

**What are the implications of the main findings?**

- Clinical aggression and ideological hostility require different responses. Pharmacological treatment addresses the clinical driver of aggression, whereas ideological hostility calls for structural measures including institutional fairness, cooperative intergroup contact, identity-complexity education, and platform redesign, which are given analytical weight equal to that of neurochemical and cognitive interventions.
- Nine study designs with explicit falsification criteria and imaging specifications are proposed as guidelines for future preregistered tests across reward recalibration, platform friction, identity-complexity training, sacred-value priming, intergroup contact, digital habituation, pride facets with contemplative practice, institutional legitimacy, and the direct comparison of prestige-derived and dominance-derived rank.

**Abstract:**

Human societies depend on prestige hierarchies that confer status on demonstrated competence, alongside the dominance hierarchies that rely on coercion and that humans share with other primates. The same neural processes that support cooperative learning under prestige are hypothesized to participate in rivalry, polarization, and ideological hostility when their operating environments depart from those in which they evolved. This selective narrative review proposes a candidate integrative framework, the Hypothesized Neural Integration Model of Prestige and Pride, drawing on converging but largely indirect evidence from affective neuroscience, neuroeconomics, evolutionary psychology, and social neuroscience. The framework groups five categories of functionally pluripotent processing, each understood as engaging nodes within overlapping large-scale brain networks rather than as a localized regional mechanism: valuation-related processing implicating orbitofrontal and ventromedial prefrontal cortex, reward-learning signals indexed in the ventral striatum, self-referential and identity-relevant processing engaging medial prefrontal cortex within the default-mode network, threat- and salience-related processing involving the amygdala and anterior cingulate cortex within the salience network, and neuroendocrine output regulated through the hypothalamic–pituitary–adrenal axis and modulated by gonadal and neuropeptide systems. Consistent with Poldrack on reverse inference and with Marr on levels of analysis, the framework is presented at an integrative implementational level with candidate algorithmic mappings specified for each category, not as a computational specification of the constructs themselves, and treats high-level constructs such as prestige, status, and sacred value as emergent phenomena arising from dynamic network interactions, bodily states, cultural contexts, and historical contingencies rather than as properties localized to single regions. Because few neuroimaging paradigms isolate freely conferred deference from generic social rank, claims specific to prestige are deliberately restricted throughout, and the evidence base is presented as one concerning social rank unless a paradigm operationalizes prestige directly. Material ownership, sacred values, religious belief, and digital prestige metrics are treated as candidate domains of application, with explicit separation of direct neural evidence, behavioral evidence, and theoretical extrapolation. Symbolic escalation in consumer markets, sacred-value absolutism, and amplified social feedback on digital platforms are interpreted as candidate environmental amplifiers of ancestral status mechanisms rather than demonstrated causal pathways. Nine operational study designs are proposed to guide future preregistered tests, including one designed to establish whether a prestige-specific neural claim can be made at all. Intervention strategies are mapped to candidate processing and network targets using an explicit evidence-grading scheme, with structural and institutional measures weighted equally alongside cognitive and contemplative ones. The framework relies on correlational neuroimaging subject to reverse-inference constraints, draws primarily from samples from Western, educated, industrialized, rich, and democratic populations, and should be evaluated as a hypothesis-generating account rather than as a confirmed neural architecture.

## 1. Introduction

### 1.1. Why Status Matters Biologically

Human societies are organized hierarchically. Unlike many non-human primates, whose social structures rely primarily on dominance upheld through coercion, human groups have also developed systems based on prestige, in which individuals are voluntarily accorded status because they are perceived as competent, knowledgeable, or resourceful [1]. The two mechanisms coexist rather than displacing one another [2]; the distinctive human trajectory involves the addition of prestige rather than the elimination of dominance. Prestige offers adaptive advantages, including access to mates, coalition support, influence over collective decisions, and improved survival prospects, and its emergence is associated with reduced reliance on costly physical confrontation. Pride functions as a regulatory emotion that reinforces status-elevating behavior and signals reliability to others [3].

### 1.2. The Central Thesis and What It Does Not Assert

This review proposes that prestige pursuit, material ownership, religious devotion, ideological commitment, digital status seeking, and self-sacrifice can be examined within a single organizing framework. The framework links valuation-related processing involving orbitofrontal and ventromedial prefrontal cortex, reward-learning signals in the ventral striatum, self-referential and identity-relevant processing within medial prefrontal cortex of the default-mode network, threat- and salience-related processing involving the amygdala and anterior cingulate cortex within the salience network, and neuroendocrine output regulated through the hypothalamic–pituitary–adrenal axis.

**Figure 1:**
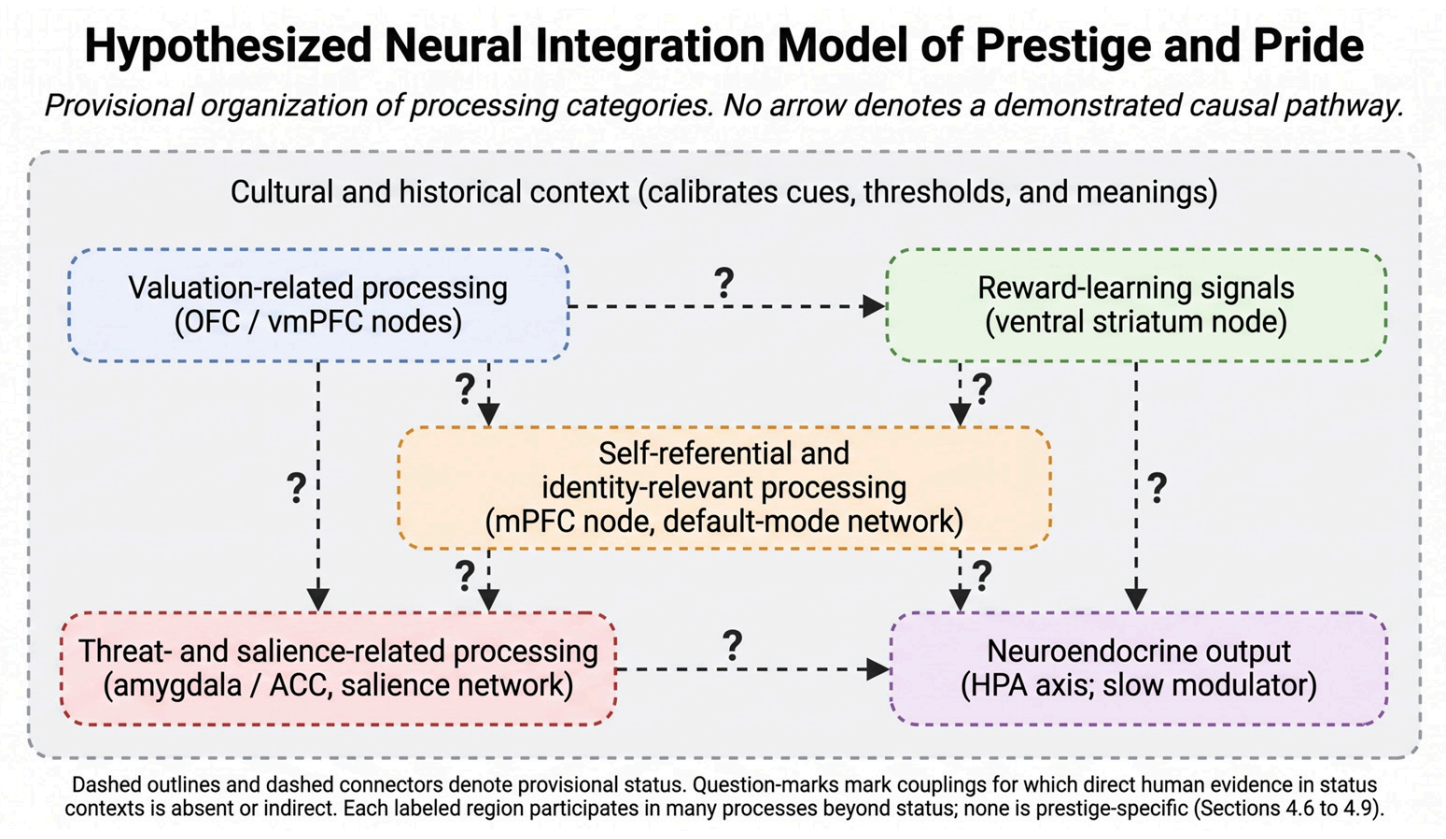
Hypothesized Neural Integration Model of Prestige and Pride. Dashed outlines and dashed connectors indicate that every element of the schematic is provisional. Connectors represent candidate functional couplings among five categories of processing whose anatomical substrates are nodes within overlapping large-scale networks (Section 4.9): valuation-related processing involving OFC and vmPFC; reward-learning signals indexed in the ventral striatum; self-referential and identity-relevant processing engaging mPFC within the default-mode network; threat- and salience-related processing involving amygdala and ACC within the salience network; and neuroendocrine output regulated through the HPA axis and modulated by gonadal and neuropeptide signaling. Question marks identify couplings for which direct human evidence in status contexts is absent or indirect. Cultural context is drawn as an enclosing frame rather than as an input node, because it calibrates which cues activate these processes and what their states mean (Section 4.9 and Section 13.3). No arrow in this figure denotes a demonstrated causal pathway, and no direction of influence should be read from arrowhead placement. Each region participates in many functions beyond status processing; none is prestige-specific.

Three disclaimers govern every claim that follows. First, no region named here is dedicated to the construct with which it is associated; each participates in many processes beyond status and is a node within extensive, overlapping, context-sensitive networks whose interactions, rather than isolated regional activity, generate the social cognition under discussion (Section 4.9). Second, the framework is positioned at the implementational level, with candidate algorithmic mappings (Section 4.7 and Section 4.9), rather than as a computational-level account of what prestige or sacred value is. Third, empirical support varies sharply across domains: direct neuroimaging evidence substantiates social rank, material ownership, and sacred-value processing, whereas prestige in the strict sense of freely conferred deference, digital prestige, and ideological hostility rest on behavioral evidence supplemented by theoretical extrapolation to neural mechanisms. When these processes operate within adaptive limits they support cooperation, innovation, and organized social structure. Environmental conditions that intensify them through symbolic escalation, sacred-value absolutism, identity fusion, or intense digital social feedback may contribute to rivalry, discrimination, and collective hostility. The account is probabilistic and modulating rather than deterministic. Figure 1 presents the framework as a conceptual schematic.

### 1.3. Aim, Scope, and Reader’s Guide

The objective is to synthesize dispersed evidence from neuroscience, evolutionary psychology, neuroeconomics, and social science into an integrated framework linking prestige, pride, and belief to candidate intervention targets. The scope is confined in three respects. The review concerns broadly conserved candidate neurobiological substrates and their culturally calibrated recruitment in non-clinical populations rather than individual psychopathology; clinical aggression is treated separately in Section 9. Policy implications are illustrative rather than prescriptive. The empirical evidence originates primarily from Western, educated, industrialized, rich, and democratic populations, and extrapolation beyond those samples is treated as provisional throughout.

The argument proceeds in four movements, and readers whose interest is confined to one may read it independently. Section 3 and Section 4 establish the conceptual distinctions, the processing framework, its methodological constraints, and its candidate computational mappings. Section 5, Section 6, Section 7 and Section 8 apply the framework to four empirical domains. Section 9, Section 10 and Section 11 separate clinical aggression from ideological hostility and map candidate interventions onto processing targets. Section 12 and Section 13 convert the framework into testable designs and state its limitations. The interdependence of these movements is the substantive claim of the review: an integrative framework that does not connect mechanisms to interventions and to falsification cannot be assessed as having marginal explanatory value, which is the standard set in Section 13.5.

## 2. Materials and Methods

### 2.1. Search Strategy

This is a selective narrative review rather than a systematic review. Literature was identified through structured, non-exhaustive searches of PubMed, PsycINFO, Web of Science, and Google Scholar conducted between January 2023 and February 2026, supplemented by reference-list screening and forward citation tracking of foundational studies. Search terms combined neural-system descriptors (orbitofrontal cortex, ventral striatum, medial prefrontal cortex, amygdala, hypothalamic–pituitary–adrenal axis) with construct descriptors (prestige, dominance, pride, sacred values, identity fusion, social comparison, social media reward, intergroup threat). Representative Boolean queries included (“prestige” OR “social rank”) AND (“ventral striatum” OR “vmPFC”), (“sacred values” OR “deontic processing”) AND (“fMRI” OR “neuroimaging”), and (“social media” OR “online platform”) AND (“nucleus accumbens” OR “reward”). Because the review is selective rather than systematic, no PRISMA flow diagram, exhaustive Boolean search, or duplicate screening procedure is reported.

### 2.2. Inclusion and Exclusion Criteria

Records were retained when they met all four of the following criteria: peer-reviewed publication; a human sample, or an animal model that provides mechanistic clarification of a human finding that is itself cited; an outcome measure operationalizing at least one of the constructs listed in Table 1; and sufficient methodological reporting to permit classification of evidence type under Section 2.3. Records were excluded when the construct was inferred only from author interpretation without a corresponding measure, when the report was a conference abstract or preprint without peer review, when neuroimaging results were reported without a stated correction for multiple comparisons, and when a finding duplicated an earlier report of the same sample. Priority within the retained set was given to meta-analyses, to studies with independent replication, and to studies whose paradigm operationalized the construct directly rather than by proxy. Supplementary Table S1 lists every study that supports a neural or behavioral claim in Section 3, Section 4, Section 5, Section 6, Section 7, Section 8, Section 9, Section 10 and Section 11, with its sample size, paradigm, replication status, and principal limitation, so that readers can audit the selection rather than accept it.

### 2.3. Evidence Categorization

Rather than a single ordinal grade, evidence for each construct is documented across three dimensions: direct human neuroimaging evidence using a paradigm designed for the construct; behavioral or psychophysiological evidence; and inference or extrapolation from related but non-identical paradigms. Table 1 applies explicit rules to the third dimension. Inference is rated Low when at least three independent direct neuroimaging studies operationalize the construct itself and at least one independent replication or meta-analysis exists; Moderate when direct studies exist but number fewer than three, or when the paradigm operationalizes an adjacent construct such as social rank in place of prestige; and High when no paradigm operationalizes the construct directly, when the evidence derives from a single sample, or when a population-specific finding would have to be generalized to a different population to support the claim. Intervention evidence in Table 3 is graded separately under the scheme defined in Section 11.5.

### 2.4. Framework Construction

The framework was constructed by identifying categories of functionally pluripotent processing that recur across paradigms of prestige, rank, ownership, sacred value, and digital validation. These categories are organizational descriptors for processes involving nodes within overlapping large-scale networks (Section 4.9) rather than functionally distinct or anatomically separable systems. Each category was included on three conditions: substantial evidence in non-prestige paradigms, recurrence across multiple status-related domains, and consistency with anatomical and functional relationships established in non-prestige research [4,5,6]. Section 4.6 states why the count is five, what would justify four or six, and which observations would revise it. Following Poldrack and Marr [7], the framework is presented at the level of converging large-scale network engagement rather than as a mapping of regions onto constructs. It is a synthetic proposal designed to organize dispersed findings and generate testable predictions (Section 12), not a confirmed causal architecture.

### 2.5. Authorship, Selection Bias, and Mitigation Procedures

This review has a single author, whose primary disciplinary background is pharmaceutical and regulatory science and whose engagement with affective and social neuroscience is that of a synthesizing outsider. The combination is deliberate, pairing a structural and policy lens with the neuroscience literature on which the framework draws, but it is also a source of bias that a reader is entitled to weigh. Sentences in an earlier version of this section referred to multiple investigators; that phrasing was an editing error and has been corrected.

Selection bias is the principal threat to any narrative synthesis, and generic assurances do not address it. Four procedures were applied and are listed in Supplementary Table S1. First, for every construct in Table 1 the search was rerun with terms designed to surface disconfirming results, pairing each construct with “null,” “failed replication,” “no effect,” and “reanalysis,” and every qualifying record was cited in the relevant section rather than in the limitations; this procedure brought the multivariate reanalyzes of social and physical pain overlap (Section 4.4), the meta-analytic weakening of the dual-hormone hypothesis and the registered replication failure for oxytocin (Section 4.5), the candidate-gene replication failures (Section 10.1), and the skeptical growth-mindset meta-analysis (Section 11.1) into the text. Second, no construct was retained in Table 1 on the strength of a single study without the entry recording that fact. Third, the principal alternative frameworks are assessed in Section 13.5 against the specific predictions they can and cannot generate rather than merely named. Fourth, the designs in Section 12 were written before the evidence was weighed, and each carries a falsification criterion that would count against the framework. Residual bias remains, and the synthesis should be treated as hypothesis-generating rather than as a consensus statement.

## 3. Conceptual Foundations

### 3.1. Dominance, Prestige, and a Scope Restriction

Cheng, Tracy, and Henrich [2] empirically distinguished dominance and prestige as two routes to social rank. Dominance relies on intimidation, coercion, and force; prestige relies on competence and voluntary deference. The evidence base for this distinction is primarily behavioral.

A scope restriction follows, and it is stronger than the qualification offered in earlier versions of this work. Almost no human neuroimaging paradigm isolates freely conferred deference from generic social rank. The studies most often cited in support of a neurobiology of prestige, including hierarchy-viewing and competitive-ranking designs [8], rank-learning designs [9], and their computational successors [10], operationalize position within a hierarchy rather than the voluntary conferral of status based on perceived competence. Prestige and dominance are distinguishable behaviorally and have partly different correlates, so evidence about rank cannot be assumed to transfer. Accordingly, this review reports the evidence as evidence about social rank wherever the paradigm measured rank, reserves the term prestige for claims resting on paradigms that operationalize voluntary deference, and treats any statement connecting the neural literature specifically to prestige as an extrapolation requiring the direct test set out as Design 9 in Section 12.3. Readers should regard the neural sections of this review as a framework for status processing broadly construed, within which prestige is a hypothesized but not yet isolated special case.

### 3.2. Neuroendocrine Correlates of Rank: Beyond a Single-Hormone Account

Sapolsky [11] documented chronically elevated glucocorticoid levels in low-ranking primates, linking status perception to stress physiology. That work concerns hierarchy in general and, by itself, does not establish rank-specific endocrine patterns in humans. In humans, the more informative literature concerns the joint action of gonadal and adrenal signals. Mehta and Josephs [12] proposed the dual-hormone hypothesis, under which testosterone is positively associated with dominance behavior only when cortisol is low, with the relationship blocked or reversed when cortisol is high. This proposal specifies an interaction rather than a main effect and thereby offers a more precise mechanism for the dominance and prestige distinction than an account resting solely on the hypothalamic–pituitary–adrenal axis. The interaction is not securely established. A meta-analysis of 33 studies reported a small, significant interaction between testosterone and cortisol, with substantial heterogeneity in direction and magnitude, stronger for explicit indices of status than for behavioral categories such as aggression, and stronger in male than in female samples [13]. Wu and colleagues [14] likewise found that victory in a social competition increased preference for higher-status products, even when anticipated testosterone reactivity was absent, suggesting that competition-induced status motivation can operate independently of the neuroendocrine markers usually invoked. The dual-hormone account is therefore adopted here as the most specific available neuroendocrine hypothesis for status dynamics and is simultaneously flagged as one with a mixed replication record.

### 3.3. Authentic Versus Hubristic Pride

Tracy and Robins [3] distinguished authentic pride, which arises from specific accomplishments, from hubristic pride, characterized by pervasive self-enhancement, on factor-analytic and behavioral grounds. Authentic pride is associated with perseverance, prosocial leadership, and long-term goal pursuit; hubristic pride correlates with narcissism, aggression, and fragile self-esteem [15]. These are empirically validated psychological constructs. The proposal that authentic pride engages medial and dorsolateral prefrontal control processes, while hubristic pride engages reward circuits with diminished prefrontal regulation, has not been adequately replicated and is treated here as a conjecture, tested in Design 7 of Section 12.3.

### 3.4. Identity Fusion Versus Social Identification

Standard social identification [16] involves cognitive recognition of group membership without embodied merger. Identity fusion [17] describes a visceral sense of oneness in which personal and group identities become functionally equivalent, with fused individuals reporting willingness to fight or die for the group. The construct is supported primarily by behavioral measures. Direct neuroimaging of identity fusion is limited. Cikara, Botvinick, and Fiske [18] examined intergroup competition and harm rather than fusion. Hamid and colleagues [19] and Pretus and colleagues [20] reported fMRI findings in radicalized samples showing altered activity in the dorsolateral prefrontal cortex and the inferior frontal gyrus when participants expressed willingness to fight and die for sacred relative to non-sacred values, consistent with reduced cost–benefit deliberation under sacred framing. These results are the most direct neural evidence relevant to fusion-adjacent phenomena, but they concern sacred-value processing in populations selected for ideological extremity and should not be generalized to identity fusion in unselected samples. Identity fusion is therefore supported far more strongly by behavioral than by direct neural evidence.

### 3.5. Sacred Values and Symbolic Goods

Sacred values are non-negotiable moral commitments that resist material trade-offs [21]. Berns and colleagues [22] reported that sacred political statements are associated with increased activity in left temporoparietal junction and ventrolateral prefrontal cortex, regions implicated in semantic rule retrieval, consistent with deontic rather than utilitarian processing. Symbolic goods derive worth from social status and narrative significance rather than practical utility, and scarcity amplifies symbolic value [23]; contemporary meta-analytic work on the neural correlates of subjective value provides the current reference point for how such contextual modifiers enter valuation [24]. Sacred value is theoretically distinct from religious belief and from secular ideology, although a sacred value may originate in any of these. This review maintains the distinction and does not generalize sacred-value findings to all religious or ideological commitments.

Table 1 reports the evidence type for each construct along three dimensions: direct neural evidence, behavioral evidence, and inference or extrapolation, rated by the explicit rules stated in Section 2.3.

**Table 1:** Evidence types for each construct along three dimensions (direct neural, behavioral, inference or extrapolation).

Construct	Direct Neural Evidence	Behavioral Evidence	Inference/Extrapolation (Rated per Section 2.3)	Key References
Social hierarchy/rank	Yes (vmPFC, ventral striatum, mPFC, amygdala during rank viewing, rank learning, and status gain; multiple independent samples)	Yes (status-comparison tasks)	Low: the paradigms operationalize the construct directly; 3 or more independent direct studies plus computational replication	[1,2,8,9,10]
Prestige (voluntary deference)	No paradigm isolates prestige from generic rank	Yes (behavioral distinction from dominance)	High: no direct operationalization; every neural claim is transferred from rank paradigms (Section 3.1)	[1,2]
Dominance	Limited human neuroimaging specific to dominance rather than threat; animal hierarchy work informative but not directly transferable	Yes (prestige–dominance scale; aggression tasks)	Moderate: fewer than 3 direct human studies; endocrine mechanism contested	[2,11,12,13]
Authentic pride	Not established with adequate replication	Yes (pride scales; behavioral correlates)	High: no direct operationalization; neural separation from hubristic pride is conjectural	[3,15]
Hubristic pride	Not established with adequate replication	Yes (pride scales; narcissism inventories)	High: reward-circuit and reduced-regulation account is theoretical	[3,15]
Material ownership	Yes (mPFC during self-object association; OFC modulation by labels; multiple independent samples)	Yes (endowment effect)	Low: 3 or more independent direct studies; effects replicated across paradigms	[25,26,27,28]
Sacred values	Yes (TPJ and vlPFC rule and deontic processing; altered dlPFC, IFG, and parietal cost-consequence processing in radicalized samples)	Yes (taboo trade-off paradigms)	Moderate: direct paradigms exist but are few, and the strongest are population-specific	[19,20,21,22]
Identity fusion	Limited; Refs. [19,20] give partial fusion-adjacent evidence in radicalized samples. Ref. [18] concerns intergroup competition and harm, not fusion	Yes (verbal and pictorial fusion scales; sacrifice tasks)	High: population-specific findings would require generalization to unselected samples	[17,19,20]
Digital prestige (likes and peer feedback)	Limited; primarily a single adolescent fMRI study in a simulated platform	Yes (engagement metrics; survey work; field experiments)	High: single sample; extrapolation from adolescent peer-feedback fMRI to adult algorithmic systems is substantial	[29,30,31,32]
Ideological hostility/out-group animosity	Partial (dorsal ACC and anterior insula in social exclusion paradigms; intergroup harm and competition activations; out-group threat amygdala effects vary by appraisal)	Yes (out-group animosity engagement; outrage; survey work; field experiments)	High: no direct operationalization; the step from neural threat circuitry to ideological hostility is inferential	[18,30,33,34,35,36,37,38]

Direct neural evidence is restricted to studies that operationalize the construct in question; partial entries indicate that related but distinct paradigms are available. Inference and extrapolation are rated by the explicit criteria in Section 2.3, which key on the number of direct neuroimaging studies, the presence of independent replication or meta-analysis, and whether a population-specific finding must be generalized to support the claim. Functional plurality is assumed throughout; none of the regions named is prestige-specific (Section 4.8). Study-level detail, including sample sizes and replication status, is given in Supplementary Table S1.

## 4. Hypothesized Neural Integration Framework

The framework groups five categories of functionally pluripotent processing, each supported by interconnected anatomical substrates. The categories are presented as process descriptions rather than region-function labels to avoid the reverse-inference error described by Poldrack [39] and elaborated in Section 4.8. The regions named are nodes within large-scale networks (Section 4.9), and the framework’s operating level is situated within Marr’s [7] tripartite scheme in Section 4.9. Each region participates in many functions beyond status-related processing. Connections among the categories are candidate functional couplings, not demonstrated causal pathways. A terminological convention is applied without exception from this point forward: the five categories are referred to as processing categories, never as systems; the phrase valuation-related processing is used throughout in place of valuation system; and reward, default-mode, and salience are described as networks in the sense established by resting-state and co-activation research, not as synonyms for the processing categories. Figure 1 presents the framework as a provisional schematic. Boundary and null evidence are stated within each subsection.

### 4.1. Valuation-Related Processing Engaging OFC and vmPFC

Orbitofrontal and ventromedial prefrontal cortex are engaged by tasks indexing relative value, with activity correlating with the integration of reward expectation, social information, and memory [4,5], a pattern confirmed by coordinate-based meta-analysis of subjective value across paradigms [24]. The claim is that valuation-related processing reliably engages these regions, not that valuation is a localized function of OFC or vmPFC. As Poldrack [39] observed, inferring a specific high-level construct from regional activity is a reverse inference error; vmPFC is also engaged by self-referential thought, moral judgment, and future planning, and is a component of the default-mode and reward networks (Section 4.9). In status-relevant contexts, OFC and vmPFC engagement appears to reflect how cultural learning recalibrates reward priorities: labeling a product as expensive increases perceived pleasantness and vmPFC activation even when the product is materially identical to a cheaper alternative [25]. Read cautiously, these findings support participation of OFC and vmPFC in contextual valuation influenced by status-related manipulations rather than a specialized prestige module. Boundary note: although contextual labeling effects on vmPFC are reproducible, their magnitude varies across stimulus classes, samples, and paradigms, and not every status-relevant manipulation engages vmPFC.

### 4.2. Reward-Learning Signals Indexed in the Ventral Striatum

The mesolimbic dopamine system, principally ventral striatum and nucleus accumbens, encodes reward prediction error, the discrepancy between expected and received reward [40]. Berridge and Robinson [41] distinguished hedonic liking from motivational wanting, emphasizing the role of dopamine in the attribution of incentive salience. Acquisition of high-status objects or recognition, in the absence of prestige hierarchies, correlates with increased ventral striatal activation [8]. Prediction error is a candidate but not exclusive mechanism linking status processing, material consumption, sacred-value reinforcement, and digital validation. Whether identical prediction-error signals carry equivalent functional significance across these domains is unresolved; direct comparisons indicate that ventral striatal prediction-error responses are largely domain-general rather than socially specialized [42], which weakens rather than strengthens any inference from striatal activation to a status-specific process. Habituation through prediction-error adaptation is consistent with theories of hedonic adaptation and escalating consumption [43], but functional equivalence across content domains remains a theoretical proposition.

### 4.3. Self-Referential and Identity-Relevant Processing Engaging mPFC

The medial prefrontal cortex, particularly its dorsomedial and ventromedial subdivisions, is consistently engaged by self-referential processing [6] and is a central node of the cortical default-mode network (Section 4.9). The claim is that self- and identity-relevant content reliably engages the mPFC, together with associated default-mode nodes, not that the mPFC is the mechanism for identity integration. Prestige objects, beliefs, and group memberships may become linked to self-schema through encoding processes that engage mPFC; the inference from regional activation to a high-level construct such as identity integration must be resisted, as Poldrack [39] argued, because the same activation is observed during mentalizing, autobiographical recall, prospection, social inference, and emotion regulation. Kim and Johnson [26] showed that personal ownership enhances autobiographical encoding. Tamir and Mitchell [44] showed that disclosing information about oneself is intrinsically rewarding and engages the mPFC alongside mesolimbic reward circuits, providing the most direct evidence for coupling between self-related and reward-related processing. Kaplan, Gimbel, and Harris [45] reported that challenges to strongly held political beliefs in 40 self-identified U.S. liberal participants increased mPFC activation, which suggests that some strongly held beliefs are encoded as extensions of the self in that population and should not be generalized to all sacred or religious commitments without studies spanning diverse ideological and cultural samples. Rank-learning work adds an important qualification: mPFC engagement during hierarchy learning is selective for updating knowledge about one’s own hierarchy rather than another people, and coding of rank itself appears in amygdala and hippocampus even when the task does not require it [9]. The mPFC contribution is therefore best described as participation of a multifunctional default-mode node in self-relevant integration.

### 4.4. Threat- and Salience-Related Processing Implicating Amygdala and ACC

Amygdala and anterior cingulate cortex participate in threat detection, salience processing, and conflict monitoring in social–evaluative contexts. The amygdala is not solely a threat detector; it processes salience, relevance, and learning more broadly, and codes rank information in hierarchy-learning tasks [9]. In status-related studies, the amygdala is often, though not uniformly, engaged by status threats, social–evaluative challenges, and out-group cues. Intergroup competition and harm paradigms have been associated with amygdala engagement [18], but this association should not be generalized to all status or out-group contexts. The anterior cingulate cortex monitors conflict between competing representations and allocates control [46]; in status and belief contexts, ACC activation has been linked to detection of reputational inconsistency and to signaling of conflict when identity-relevant information is challenged.

The relationship between social and physical pain requires correction relative to earlier accounts. Eisenberger and Lieberman [33] reported that social exclusion engages dorsal ACC and anterior insula in patterns overlapping those observed during physical pain, and this observation was historically influential. Subsequent multivariate work has undermined the strong reading. Wager and colleagues [47] identified a distributed multivariate signature specific to physical pain, and Woo and colleagues [38] showed that pattern classifiers discriminate physical pain and social rejection from their respective controls in out-of-sample participants, that each classifier performs at chance on the other condition, and that pain-related and rejection-related representations are uncorrelated within regions previously thought to encode pain affect, including dorsal ACC. The correct statement is therefore that social exclusion and physical pain engage overlapping regions at the level of univariate activation while carrying separable multivariate representations within those regions. The claim that reputational and physical threats are functionally continuous is not supported and has been removed from this framework. What survives is weaker yet still useful: social–evaluative threat recruits the same salience-network nodes as physical threat, consistent with shared alerting and control functions rather than a shared representation of pain. Not every exposure to an out-group or status challenge produces amygdala activation; effects depend on threat appraisal, individual differences, and task design.

### 4.5. Neuroendocrine Output: HPA-Axis Regulation and Neuropeptide Modulation

The hypothalamic–pituitary–adrenal axis governs cortisol secretion in response to social evaluation and variation in social status. Dickerson and Kemeny [48] demonstrated, using meta-analysis, that social–evaluative threat reliably elevates cortisol. Sapolsky [11] observed chronically elevated glucocorticoids in subordinate primates. Sustained instability of social status, whether driven by economic disparity, identity threat, or persistent social comparison, is associated with sustained cortisol elevation and, in several experimental paradigms, with cognitive rigidity and defensive behavior. As set out in Section 3.2, the informative human account is not adrenal output alone but its interaction with gonadal signaling under the dual-hormone hypothesis [12], with the caveat that meta-analytic support is small and heterogeneous [13].

Oxytocin warrants explicit treatment because it bears directly on the in-group and out-group dynamics central to this review, and its omission from earlier versions of this work was a substantive gap. De Dreu and colleagues [49] reported in double-blind, placebo-controlled experiments that intranasal oxytocin promoted in-group trust and cooperation, and defensive but not offensive aggression toward competing out-groups, a pattern the authors described as tend-and-protect. Ditzen and colleagues [50] reported that intranasal oxytocin increased positive communication behavior and reduced cortisol during couple conflict, suggesting that the peptide modulates social–evaluative stress rather than a general prosocial agent. Two constraints apply. First, the parochial pattern means that oxytocin cannot be read as a candidate intervention target for reducing intergroup hostility; if anything, the reported profile predicts that enhancing in-group bonding without altering the intergroup structure would sharpen group boundaries. Second, the intranasal oxytocin literature has a weak replication record. A high-powered registered replication by several of the original authors found no effect of oxytocin on trusting behavior under the conditions of the original demonstration [51]. Oxytocin is therefore included here as a mechanistically relevant modulator with a contested evidence base, not as an established component of the framework, and no intervention in Section 11 targets it.

Neuroendocrine output is retained as a separate category rather than folded into threat- and salience-related processing for the reasons given in Section 4.6.

### 4.6. Why Five Categories, and What Would Revise the Number

A taxonomy with an unjustified cardinality invites the objection that the number was chosen for narrative convenience. Three principles fix the count at five in the present formulation, and each identifies the observation that would change it.

The first principle is separation by computational role. A category is retained only if it corresponds to a distinguishable class of signal in the sense of Section 4.7: value comparison, prediction error, self-relevance and belief updating, precision or salience allocation, and slow systemic gain control. Two categories collapse into one if no experimental dissociation of their signals survives, which is why valuation-related processing and reward-learning signals are held apart, since value comparison at decision and prediction error at outcome are dissociable in time and in parametric response despite sharing anatomy.

The second principle is separation by timescale and mode of action, which is the specific justification for retaining neuroendocrine output as a distinct category rather than merging it with threat- and salience-related processing. The objection is well-founded anatomically, since the amygdala drives hypothalamic activation, and glucocorticoids feed back onto amygdala function. The categories nevertheless differ on three counts. Their timescales differ by orders of magnitude: salience-network responses unfold over hundreds of milliseconds to seconds, while cortisol responses unfold over minutes to hours, and chronic elevation unfolds over weeks to years. Their modes of action differ, since salience processing is phasic and event-locked, whereas glucocorticoid action is tonic and systemic, altering the gain of many circuits rather than signaling a specific event. Their measurement and intervention profiles differ, as shown in Table 3. Merging them would predict that any manipulation altering phasic threat responses would also alter cortisol output, and vice versa, which is contradicted by dissociations between subjective threat appraisal and cortisol reactivity. The categories would merge if a manipulation moved both measures with a common dose–response profile and no dissociable component.

The third principle is that the taxonomy is provisional and subordinate to a signal-level account. Active inference offers an alternative and arguably more informative decomposition in which the relevant distinctions are between prediction error, prior updating, and precision or arousal rather than between anatomical categories [52], yielding three levels that would subsume the present taxonomy. It is not adopted as the primary organization because most of the literature reviewed reports regional and network findings that cannot be reanalyzed at the signal level without the original data, and a signal-level framework unconnected to the existing evidence base would not be testable in the near term. Section 4.7 states what each of the five categories would correspond to under such an account, which is the bridge that makes eventual replacement possible. A demonstration that a three-signal decomposition accounts for the same phenomena with fewer free parameters would supersede the five-category organization, and this review treats that outcome as desirable rather than threatening.

### 4.7. Candidate Computational Signals for Each Category

A framework operating at the level of Marr’s algorithm must state what is being computed, not only where. This subsection specifies a candidate computational signal for each processing category, the model class within which it is formalized, and the evidence gap. None is established specifically for status contexts, and each is the hypothesis that makes the corresponding design in Section 12 quantitative rather than directional.

The framework remains closer to an integrative implementational account than to a computational theory. What Table 2 supplies is the specification needed to make it algorithmically addressable: each category now carries a named signal, a model class, and a parameter that a study could estimate.

The framework remains, on its own assessment, closer to an integrative implementational account than to a computational theory. This subsection provides the specification needed to make it algorithmically addressable: each category now carries a named signal, a model class, and a parameter that a study could estimate.

### 4.8. Reverse Inference, Replication, and the Constructs-from-Activation Fallacy

A single methodological caution governs the entire review. Poldrack [39] argued that inferring a specific cognitive process from regional activation, without independent evidence that the region is selectively engaged by that process, is a reverse-inference error. The vmPFC is engaged by value computation, self-referential thought, moral judgment, and future planning. The ventral striatum responds to food, monetary, social, humorous, and prediction-error contexts, and its prediction-error responses are domain-general [42]. The amygdala is engaged by salience, novelty, learning, and rank coding as well as by threat [9]. The mPFC is a default-mode hub participating in mentalizing, prospection, autobiographical recall, and self-related processing. The descriptors used here are process-first formulations. They denote that the named tasks reliably engage the named regions in the empirical record, not that the named regions are the mechanism for prestige, status, pride, sacred value, or identity fusion. Equating regional activation with a high-level social construct collapses participation in a process into identity with the construct, which is the specific fallacy the framework is designed to avoid.

Replicability concerns in social neuroscience are severe and are treated here as a constraint on the framework rather than as a caveat appended to it. Many influential findings have not been independently replicated with adequate power, sample sizes are frequently in the range of 15 to 30 participants, and effect sizes are correspondingly inflated [55]. The multivariate reanalysis of social and physical pain described in Section 4.4 is an instructive case, since a widely cited overlap claim did not withstand scrutiny under a method with greater representational resolution. Every neural claim in this review should be read within these limits: regional activation indicates participation consistent with the framework, not exclusive functional specialization, and the explanatory claims operate at the level of converging network engagement and integrative description rather than region-to-construct identity.

### 4.9. Network Embedding and Levels of Explanation

The five categories are not five anatomically isolated systems. Their anatomical substrates are nodes embedded within a small number of overlapping, context-sensitive large-scale networks identified through resting-state connectivity and task co-activation [56]. Three are directly pertinent. The default-mode network encompasses medial prefrontal cortex, posterior cingulate cortex, and lateral parietal regions and is engaged by self-referential thought, mentalizing, autobiographical recall, and prospection; the mPFC node of Section 4.3 belongs to this network rather than constituting an identity-integration system in isolation [57]. The salience network, anchored in anterior insula and dorsal anterior cingulate cortex with subcortical extension to the amygdala, detects behaviorally relevant events and switches attention between internal and external stimuli; the processing described in Section 4.4 is best interpreted as activity within this network rather than as an isolated amygdala and ACC pair [58]. The reward and valuation network, comprising ventral striatum, orbitofrontal cortex, ventromedial prefrontal cortex, and dopaminergic midbrain, underpins the phenomena of Section 4.1 and Section 4.2 [4,5]. Neuroendocrine output operates as a slower modulator interacting with these cortical and subcortical networks rather than as a parallel circuit. Two consequences follow. The social cognition discussed here is plausibly generated through interactions among these networks rather than by isolated activity within any one of them, and the shorthand of five categories is a device for organizing dispersed evidence rather than an assertion of five dissociable modules.

Marr [7] identified three levels at which an information-processing system can be analyzed: the computational level, which specifies the goal and its rationale; the algorithmic level, which specifies representations and procedures; and the implementational level, which specifies the physical substrate. Section 4.1, Section 4.2, Section 4.3, Section 4.4 and Section 4.5 and the network embedding above operate predominantly at the implementational level, with the candidate algorithmic mappings of Section 4.7 indicating what the corresponding algorithmic-level description would be. The framework does not provide a computational-level specification of prestige, status, pride, sacred value, or identity fusion, and does not claim to.

A caveat about the source of this scheme is owed to readers in visual neuroscience. Marr developed the three-level analysis for vision, a domain with a well-posed computational problem, a physically specifiable input, and a criterion of success external to the observer. Social cognition has none of these features in the same form: what counts as a prestige cue or a status threat is partly constituted by the culture in which it occurs, so the computational-level question is not simply unanswered but partly normative. The three-level scheme is therefore used here as an organizing framework for keeping levels of explanation distinct, rather than as a claim that status processing is analogous in structure to visual processing or that a Marr-style computational theory of prestige is available, in the sense that one is for stereopsis.

This positioning has a substantive implication. Status, prestige, and sacred value are not properties confined within neural tissue. They are emergent phenomena arising from the dynamic interactions among neural networks, bodily states, including neuroendocrine output, cultural contexts, and historical contingencies. The cultural and historical elements are constitutive rather than supplementary, since judgments about what counts as a prestige cue, a sacred commitment, or a status threat are determined partly by socialization, institutions, and historical trajectory, with anthropological evidence documenting substantial cross-cultural variation (Section 13.3). A framework confined to the implementational level, therefore, leaves the computational specification of these constructs unresolved. The present review acknowledges this directly: the framework is an integrative organization of dispersed findings, useful for generating preregistrable predictions about network engagement and intervention targets, and it is not a substitute for a computational-level theory of what prestige or sacred value is and why human social ecologies produce these constructs in the forms observed. Research combining network-level imaging with explicit computational models of social rank and sacred-value processing, evaluated across diverse cultures, is required before the implementational description can be confidently linked to the constructs.

## 5. Evolutionary Transition of Status Systems

### 5.1. From Primate Dominance to Human Prestige

In most non-human primate species, hierarchy is maintained through physical stature, aggression, and direct coercion. Selection pressures favoring dominance hierarchies relate to immediate control over resources, and the system carries a high metabolic cost, produces instability, and requires continuous enforcement [11]. Henrich and Gil-White [1] proposed that human prestige evolved as a mechanism supporting social learning, shifting selection pressure from immediate resource acquisition toward cooperative advantage: individuals able to identify and imitate proficient group members gained benefits without the costs of physical competition. Infants aged 10 to 13 months, but not those at 8 months, recognize conflicting goals between novel agents and use relative size to predict the outcome of dominance contests [59]. Preschool children preferentially learn from individuals identified as knowledgeable rather than merely dominant [60], indicating an early developmental shift toward prestige-based social learning alongside continuing sensitivity to dominance.

### 5.2. Symbolic Prestige and Large-Scale Coordination

**Figure 2:**
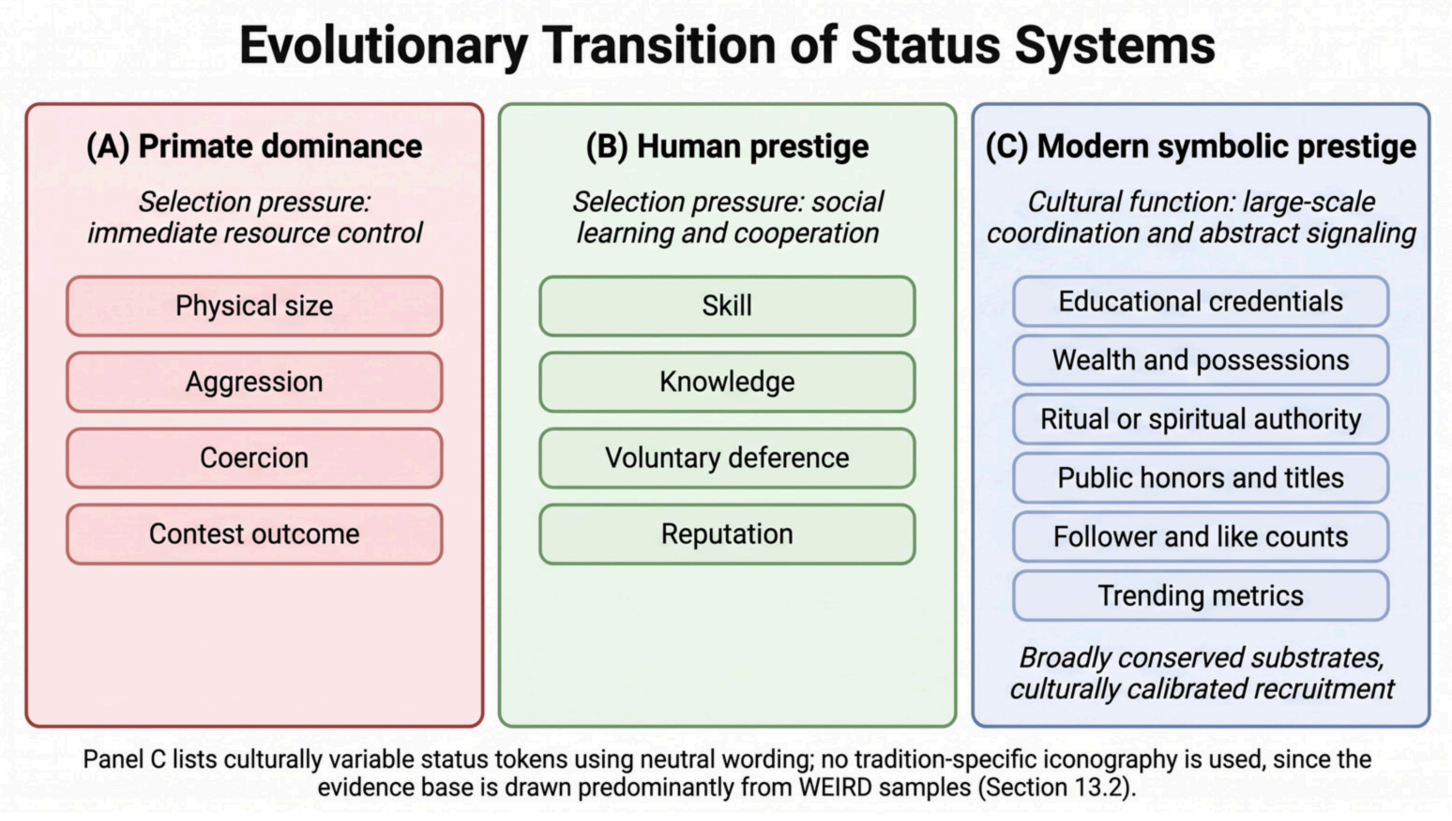
Evolutionary Transition of Status Systems. (**A**) Primate dominance hierarchies: selection pressure is immediate resource control through physical size, aggression, and coercion. (**B**) Human prestige hierarchies: selection pressure is social learning and cooperative amplification through skill, knowledge, and voluntary deference. (**C**) Modern symbolic prestige: culturally variable status tokens recruit broadly conserved but culturally calibrated neural substrates rather than a single shared architecture, and the cultural function is large-scale coordination and abstract signaling rather than direct biological selection. Panel (**C**) uses neutral wording for each token and no longer includes tradition-specific iconography, since the evidence base underlying the framework is drawn predominantly from Western, educated, industrialized, rich, and democratic samples, and the panel should not imply that any one tradition is representative of religious or spiritual authority (Section 13.3).

Gene-culture coevolution [61] posits that prestige systems emerged as symbolic complexity increased, although the relative contributions of biological selection on neural structure and of cumulative cultural change to modern symbolic prestige remain contested. Language spread reputation beyond direct observation, and institutions formalized prestige through titles, ranks, and honors. Henrich [62] argued that prestige bias accelerated cumulative cultural evolution and enabled societies to expand beyond kinship. Contemporary prestige markers are best understood as cultural elaborations operating on neural processes whose original selection pressures were ancestral; they reflect cultural function and institutional incentive rather than direct biological selection. Figure 2 illustrates the transition with that distinction in mind.

### 5.3. Boundary Conditions: When Status Systems Stabilize Versus Destabilize

Status systems are stable when they track authentic competence, remain open to revision, and distribute standing across multiple domains. They destabilize under identifiable conditions: when status is concentrated in zero-sum domains, when symbolic escalation outruns any underlying competence signal, when status becomes fused with sacred values and therefore non-negotiable, and when digital environments intensify competition through unpredictable social feedback with no natural equilibrium. These conditions are moderated by cultural tightness and looseness [63], institutional legitimacy, resource ecology, and individual differences, examined in Section 10.

### 5.4. Prestige as a Cooperative Amplifier

Status systems have more often supported large-scale cooperation than they have provoked conflict. Indirect reciprocity, which sustains cooperation through reputation, depends on status-tracking processes that reward prosocial actors with elevated standing [64]. Cultural group selection models propose that groups with efficient status allocation outcompete those without [65,66]. Prestige bias accelerates the adoption of cooperative innovations, health practices, and sustainable technologies when high-status individuals model them [62]. The prosocial function is the evolutionary norm; escalation and conflict indicate boundary violation rather than typical operation.

## 6. Ownership, Symbolism, and Identity Extension

### 6.1. The Extended Self

Ownership alters valuation through the endowment effect [67]. Self-owned items engage medial prefrontal regions associated with self-referential processing [6], and personal ownership enhances autobiographical encoding [26]. Prestige objects may therefore function as components of identity representation, consistent with the coupling between self-related and reward-related processing reported by Tamir and Mitchell [44].

### 6.2. Authenticity, Luxury, and Scarcity Signaling

Perceived authenticity influences valuation-related processing. Kirk and colleagues [27] found that labeling art as originating from a prestigious gallery increased orbitofrontal activation independently of intrinsic aesthetic quality, and when authenticity is uncertain, the anterior cingulate cortex may register inconsistency [46]. Scarcity amplifies symbolic value through rarity-based reward [23], an effect best interpreted through current models of how contextual modifiers enter a common valuation signal [24]. Brand labeling affects neural reward responses independently of objective product qualities [28], indicating that symbolic goods act as socially constructed value multipliers.

### 6.3. When Material Identity Becomes Defensive

When self-worth becomes contingent on material status markers, the proposal that reward processes habituate through prediction-error adaptation and require escalating signals is offered as a theoretical integration rather than a demonstrated mechanism. Kasser and Ryan [68] reported that strong extrinsic value orientation is associated with reduced well-being and increased anxiety. Festinger’s social comparison theory [69] describes how chronic upward comparison engages threat-related processing. Direct neural evidence linking loss aversion for identity-embedded objects to amygdala defensive responses is sparse and remains a target for empirical work. Cognitive dissonance [70] may further entrench material identity when external display conflicts with private knowledge of inadequacy.

## 7. Belief Systems and Sacred Values

This section treats religious and ideological beliefs as composite phenomena comprising belief content, ritual practice, social identity, institutional structure, and intergroup boundary marking. Sacred values are treated as a separate construct that may arise within religious or secular ideological domains. Findings within one belief domain are not extrapolated to another without qualification.

### 7.1. Belief as Identity Architecture

Belief systems developed as coordination mechanisms enabling cooperation among large numbers of unrelated individuals beyond kinship [61,71]. Neuroimaging of religious belief has reported engagement of mentalizing regions including temporoparietal junction and medial prefrontal cortex [72], and Schjoedt and colleagues [73] found that improvised personal prayer in highly religious participants engaged temporopolar cortex, medial prefrontal cortex, temporoparietal junction, and precuneus relative to formalized prayer and secular control conditions, which the authors interpreted as recruitment of social-cognition regions when the addressee is treated as a reciprocating agent. These activations align with participation of default-mode and mentalizing networks (Section 4.9) rather than with religion-specific circuitry, and they are subject to the reverse-inference constraints of Section 4.8. The samples in this literature are small, culturally narrow, and drawn overwhelmingly from Christian participants in Western settings, so the claim supported is that practices involving an imagined reciprocating agent engage mentalizing networks, not that religious cognition as such has a characteristic neural signature.

This cognitive framework may derive partly from hyperactive agency detection, an evolutionary bias toward attributing intention to agents in ambiguous environments [74]. Inzlicht and Tullett [75] reported that religious primes were associated with reduced amplitude of the error-related negativity, an event-related potential generated in anterior cingulate cortex that indexes conflict monitoring. This finding should be read with two restrictions. It concerns error monitoring within a specific task and does not demonstrate that belief in a benevolent deity reduces anxiety or physiological stress. It also rests on small-sample event-related potential work of the kind for which independent well-powered replication is generally absent in this literature [55], and no adequately powered independent replication of the religious priming effect on error-related negativity amplitude has been established. The effect is therefore reported here as suggestive rather than as a stable phenomenon. Granqvist and colleagues [76] conceptualized religion as a form of attachment; the proposal that representations of God engage parental bonding circuitry has theoretical support but limited neuroimaging evidence and should be treated as a psychological attachment hypothesis rather than a demonstrated neural overlap.

### 7.2. Sacred Values and Non-Compromise

When values attain sacred status, they are processed through deontic reasoning rather than utilitarian cost–benefit computation [22]. As noted in Section 4.3, Kaplan, Gimbel, and Harris [45] documented increased mPFC activation when strongly held political beliefs were challenged in a sample of U.S. liberals, consistent with a defensive response to identity-relevant challenge in that population. Hamid and colleagues [19] and Pretus and colleagues [20] reported reduced dorsolateral prefrontal and inferior frontal activity when participants expressed willingness to fight and die for sacred relative to non-sacred values, consistent with reduced cost–benefit deliberation under sacred framing. These two studies provide the most direct neural evidence for sacred-value processing under high-stakes commitment, and they concern samples selected for ideological extremity, which constrains the population to which any prediction derived from them applies (Section 12.3, Design 4). Sacred values are conceptually distinct from religion; treating them as identical would generalize findings across domains without empirical justification.

### 7.3. Moral Foundations and the Cross-Cultural Structure of Moral Concern

An account of sacred values that does not engage moral foundations theory omits the principal existing taxonomy of moral content, and its absence from earlier versions of this work was a gap. Haidt and Graham [77] argued that the moral domain extends well beyond harm and fairness and proposed five psychological foundations concerning harm and care, fairness and reciprocity, in-group and loyalty, authority and respect, and purity and sanctity, with subsequent development and empirical evaluation summarized by Graham and colleagues [78]. Three points of contact with the present framework matter.

First, moral foundations theory provides content, while the present framework provides process. The framework describes how a commitment comes to be processed as non-negotiable; moral foundations theory describes which commitments are likely to acquire that status and how their weighting differs across individuals and political groups. The two are complementary rather than competing, and the sacred-value literature is better read as concerning the processing consequences of moral commitments whose content moral foundations theory characterizes.

Second, the documented cross-cultural and cross-ideological heterogeneity in foundation weighting provides direct support for the constitutive role assigned to culture in Section 4.9 and Section 13.3. If the same neural processes are recruited by commitments whose content varies systematically across populations, then a species-universal implementational account cannot, by itself, explain what people treat as sacred, which is exactly the limitation this review identifies.

Third, moral foundations theory generates a discriminating prediction that the present framework does not, and this counts against the framework’s marginal utility in the sense set out in Section 13.5. Moral foundations theory predicts that the specific content of a violated commitment, rather than its sacredness, determines the pattern of moral judgment and intergroup response. A framework operating at the level of processing categories cannot derive that prediction. Where the present framework adds value is in the reverse direction: it predicts that commitments drawn from different foundations should converge on a common processing signature once they attain sacred status, which moral foundations theory does not require. Design 4 in Section 12.3 is constructed to adjudicate exactly this contrast by sampling sacred values from at least two foundations.

### 7.4. Dual-Use Nature of Belief Systems

Belief systems function in two directions. Within groups, shared sacred values increase trust, cooperation, and adherence to norms [71]. Across groups, sacred framing intensifies commitment and reduces the willingness to compromise, since conflicts portrayed as divine mandates or sacred causes are morally constrained. Critical moderators include pluralism, institutional separation of political power from sacred authority, cross-cutting identities, and the rule of law, which provides legitimate channels for grievance independent of sacred framing. Religion is rarely an isolated cause of conflict; political power, economic incentives, and territorial interests are typically intertwined with religious rhetoric, and sacred framing amplifies commitment more often than it generates conflict.

## 8. Digital Prestige and Dopaminergic Escalation

This section is organized by evidence type: direct fMRI evidence on adolescent peer feedback; behavioral evidence on moral outrage and engagement; platform-level correlational and field-experimental evidence; and the proposed neural mechanism that would connect them. The step from any one layer to a platform-level dopaminergic causal loop is large and is stated as such throughout. The section describes a hypothesized model awaiting direct empirical validation, and no claim of platform-induced dopaminergic escalation is asserted as established.

### 8.1. Variable Social Feedback and Reinforcement

Digital platforms deliver social feedback at variable and unpredictable intervals. Behavioral learning research has long characterized intermittent reinforcement as a schedule under which unpredictable feedback maintains operant behavior, and unpredictability is central to incentive-salience accounts [41]. Montag and colleagues [31] applied this framework to social media and freemium games as a theoretical integration rather than as an experimental demonstration of platform-level reinforcement. No direct evidence establishes that recommendation algorithms implement a variable-ratio schedule. The supportable claim is that digital social feedback is variable and intermittent in a manner capable of sustaining motivational engagement; equating this with a calibrated variable-ratio schedule would require platform design evidence that is not publicly available.

### 8.2. Social Validation Metrics

**Figure 3:**
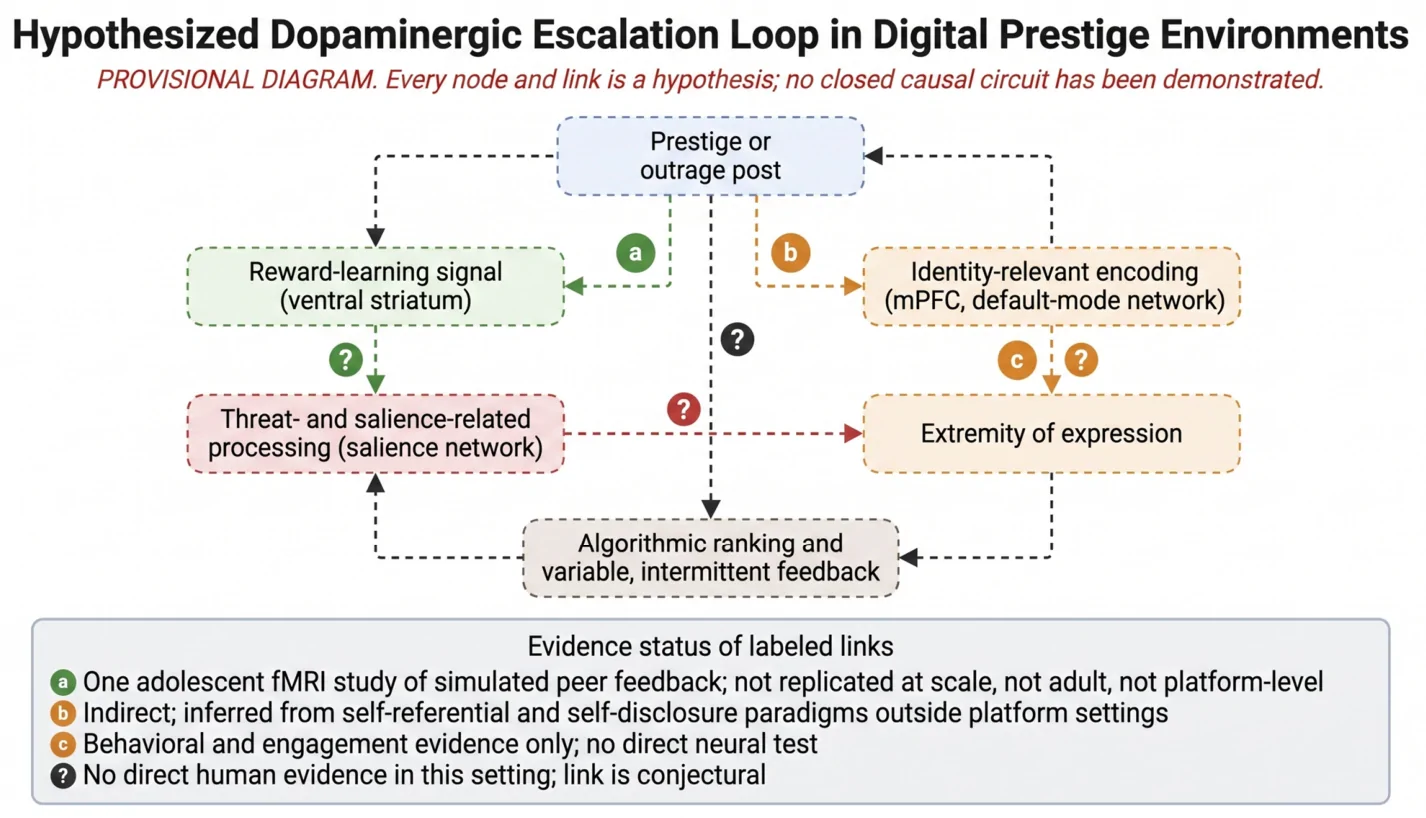
Hypothesized Dopaminergic Escalation Loop in Digital Prestige Environments. The figure is drawn as a provisional diagram: all nodes and connectors are dashed, and the evidence status of each link is marked on the link itself rather than stated only in this caption. Link a, from post to reward-learning signal, rests on a single adolescent fMRI study of simulated peer feedback [29] that is neither replicated at scale nor conducted in adults nor conducted at the platform level. Link b, from post to identity-relevant encoding, is indirect and inferred from self-referential and self-disclosure paradigms outside platform settings [6,44]. Link c, from identity-relevant encoding to extremity of expression, rests on behavioral and engagement evidence only [30,34,35,36]. Links marked with a question mark lack direct human evidence in this setting and are conjectural, including any link involving algorithmic ranking. Regional names identify empirically implicated nodes, not construct-localizing mechanisms. The causal relationship between algorithmic ranking and neural reward dynamics is unestablished, and the apparent closedness of the loop should be read as a hypothesis under test rather than a described mechanism.

Social rewards engage striatal reward pathways [32], and like-count feedback has been linked to nucleus accumbens activation in adolescents using a simulated Instagram paradigm [29]. Sherman and colleagues [29] reported that viewing photographs with many rather than few likes were associated with increased nucleus accumbens activation and greater engagement of reward, social cognition, and imitation processes. This is the most direct neuroimaging evidence available on neural responses to likes. It is a single study, in adolescents, in a simulated environment, and the extrapolation from it to adult algorithmic prestige systems is the largest single inferential step in this review. Figure 3 marks that step explicitly.

### 8.3. Identity Hardening and Polarization

Outrage functions in part as digital status signaling, since morally charged content attracts disproportionate engagement and thereby creates incentives for extreme expression [30,34]. Brady and colleagues [35] showed that moral-emotional language increased diffusion of political messages within ideological networks, and to a lesser extent between them, in a large corpus of social media communications. Rathje, Van Bavel, and van der Linden [36] reported that out-group animosity strongly predicted engagement across platforms. Cikara and Van Bavel [79] reviewed evidence that group-based emotions are processed similarly to individual emotions but amplified through identity networks. These are behavioral and engagement findings; the neural mechanism connecting them to reward processing is hypothetical. Multi-agent reinforcement-learning models of sequential social dilemmas [53] provide the formalism within which the claim that platform incentives reshape expression can be simulated and tested, a level of specification this literature currently lacks.

### 8.4. Algorithmic Ranking and Polarization

Ranking and recommendation algorithms influence the frequency and intensity of identity-reinforcing feedback [80]. Bail and colleagues [37] conducted a field experiment in which paid U.S. partisans followed opposing-ideology accounts, with increased polarization among Republicans and a non-significant trend in the same direction among Democrats, demonstrating that exposure manipulation alone does not reduce identity-protective responses and may amplify them in some populations. A systematic review of global evidence reports mixed effects of digital media on democratic outcomes, with some associations favorable in autocracies and emerging democracies and others unfavorable in established democracies [81]. The relationship is outcome- and regime-dependent rather than uniform. The dopaminergic loop connecting algorithmic ranking to neural reward processing is therefore a hypothesis supported by heterogeneous evidence, not a demonstrated mechanism, and Figure 3 is drawn to make the status of each link visible without requiring the reader to consult the caption.

## 9. Clinical Aggression Versus Ideological Hostility

### 9.1. Neurochemical Dysregulation and Treatable Aggression

When hostility is associated with clinical conditions such as psychosis, severe mood disorders, post-traumatic stress disorder, substance use, or impulse-control disorders, pharmacological intervention can reduce aggression by modulating arousal, mood stability, or paranoia. The evidence varies across drug classes and conditions. Jones and colleagues [82] meta-analyzed ten placebo-controlled trials of mood stabilizers for impulsive or repetitive aggression and found a statistically significant reduction, with notable effects for carbamazepine, oxcarbazepine, phenytoin, and lithium, although the pooled effect was attenuated and became non-significant when analysis was restricted to studies at low risk of bias. Anticonvulsant comparisons show considerable heterogeneity at the trial level [83]. Antipsychotics reduce aggression and hostility across diagnoses with modest effect sizes; the meta-analysis by van Schalkwyk and colleagues [84] included 21 trials and reported a standardized mean difference of approximately 0.29, comparable in magnitude to non-pharmacologic interventions, with no clear superiority among agents. In schizophrenia, the effect on aggression depends on the clinical pathway driving the behavior [85]. For post-traumatic stress disorder, the 2023 VA/DoD Clinical Practice Guideline (version 4.0) maintains a strong recommendation for paroxetine, sertraline, and venlafaxine, carried forward from the 2017 version 3.0 guideline, and emphasizes that pharmacotherapy reduces post-traumatic stress disorder symptoms rather than targeting hostility as such [86]. Effects vary across individuals, and treatment addresses the clinical driver rather than hostility as an isolated target.

### 9.2. Identity-Based Hostility and the Limits of Pharmacology

Where hostility is predominantly social, motivated by ideological commitment, group grievance, narratives of humiliation, or status rivalry, pharmacological intervention does not address the driver. Reducing baseline arousal might, at most, indirectly reduce reactivity. The processes described throughout this review, including reward-linked identity reinforcement, sacred-value encoding, identity fusion, and amplified social feedback, are social–ecological in origin rather than neurochemical. Effective intervention requires structural measures: institutional fairness, contact under cooperative conditions, identity-complexity education, platform redesign, and leadership in narrative framing.

### 9.3. Ethical Considerations

The distinction between clinical aggression and ideological hostility carries ethical weight. Using pharmacological agents to suppress hostility arising from legitimate grievance, political dissent, or ideological disagreement raises concerns about coercion, civil liberties, and the medicalization of political conduct. Any intervention framework must maintain a clear boundary between treating clinical pathology and engineering social compliance. The proposals in this review emphasize environmental and institutional redesign rather than pharmacological behavior modification, because the former respects individual autonomy while the latter carries a risk of authoritarian abuse.

## 10. Moderators and Protective Factors

The framework predicts variation across individuals and contexts, contingent on at least four classes of variable, each of which influences whether status processes support prosocial stability or contribute to escalation.

### 10.1. Individual Differences

Genetic variation has been associated, at the candidate-gene level, with dopaminergic transmission [87], serotonin transporter function [88], and oxytocin receptor sensitivity [89]. Several historically prominent candidate-gene findings, notably 5-HTTLPR by stress and DRD4 personality associations, have failed to replicate in large-scale studies [90], and current practice favors polygenic and gene-environment interaction approaches over single-variant claims. The candidate-gene literature is therefore cited here as historical context and is not relied upon as a mechanistic moderator. Trait-level variables, including narcissism, social dominance orientation, empathy, and threat sensitivity, modulate engagement with status cues, and gene-environment studies show that identical variants produce different outcomes depending on social ecology [91].

### 10.2. Cultural Tightness and Looseness

Gelfand and colleagues [63] showed that societies vary systematically in the strength of social norms and tolerance of deviance. Tight cultures may intensify status competition and sacred-value rigidity; loose cultures permit greater flexibility and greater instability. Honor cultures [92] are characterized by a configuration in which reputation loss triggers intense threat processing and retaliation. Collectivist and individualist orientations [93] determine whether status derives primarily from personal achievement or from fulfilling social roles.

### 10.3. Institutional Trust and Legitimacy

When institutions are perceived as fair and legitimate, chronic status threat is reduced. Transparent governance, adherence to the rule of law, and accessible grievance mechanisms may reduce sustained status-related stress by providing predictable routes to advancement and to conflict resolution. Institutional collapse or perceived corruption sustains humiliation and increases vulnerability to status-driven radicalization.

### 10.4. Identity Complexity

Roccas and Brewer [94] proposed that individuals who perceive themselves as belonging to multiple overlapping categories show reduced intergroup bias. Identity complexity obstructs full assimilation into any single group narrative, preserving cognitive flexibility and reducing the likelihood that a threat to one identity dimension compromises the entire self-concept.

## 11. Intervention Framework: Mechanisms and Evidence

**Figure 4:**
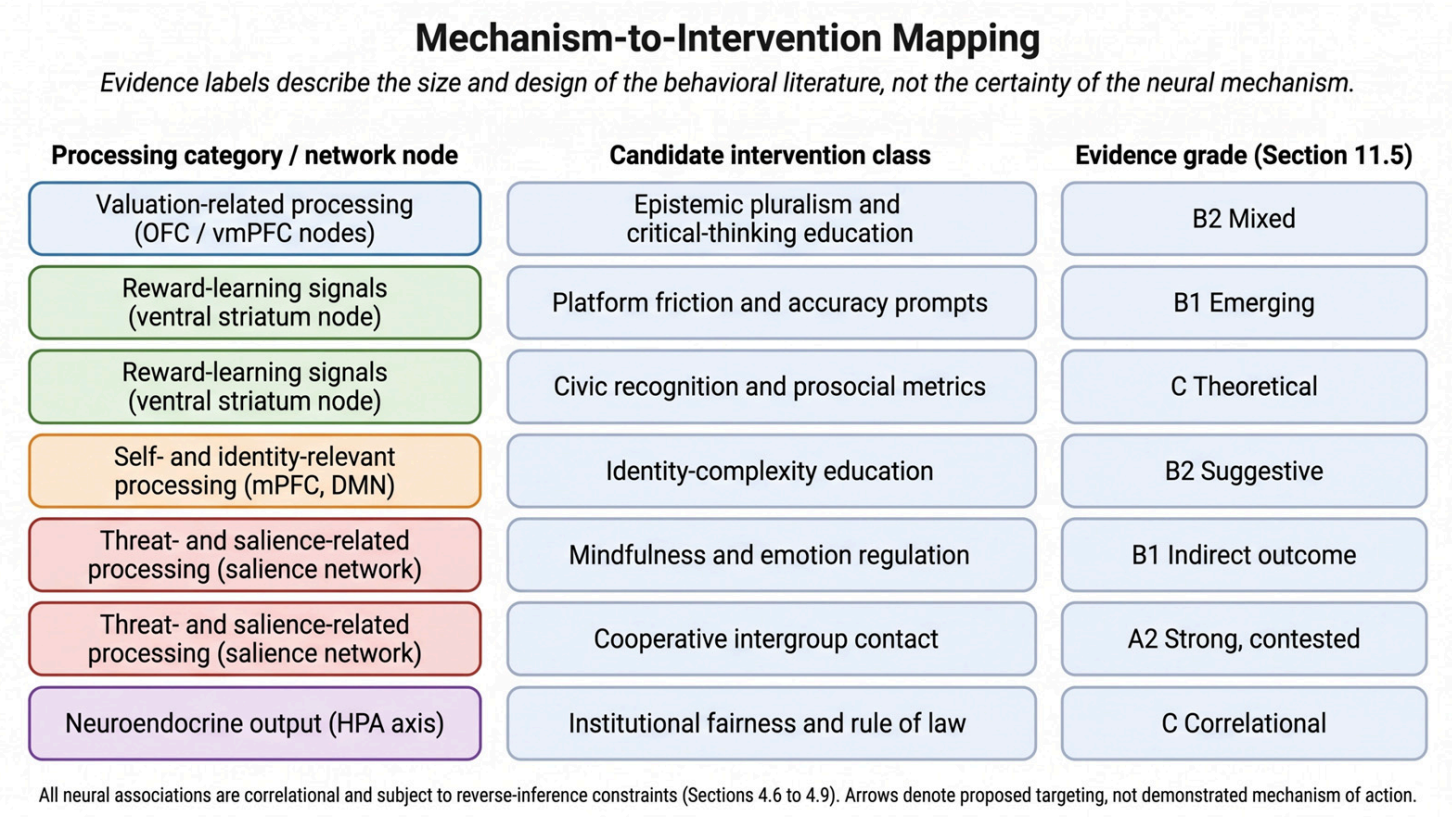
Mechanism-to-Intervention Mapping. Each processing category and network node (**left**) is linked to a candidate intervention class (**center**) and to the evidence grade defined in Section 11.5 (**right**). The left column denotes processing categories and large-scale-network nodes (Section 4.9), not localized regional mechanisms or construct-specific modules. Mindfulness and intergroup contact appear in separate rows because their evidence bases differ. All neural associations are correlational and subject to the reverse-inference constraints of Section 4.8 and Section 4.9. Evidence grades describe the size and design of the behavioral literature, not the certainty of the neural mechanism.

Interventions are classified by the processing categories and network nodes they are hypothesized to engage, not as actions on isolated neural targets. Structural and institutional interventions are given the same analytical weight as cognitive and contemplative ones, and the ordering of subsections below does not imply a hierarchy of importance; on the evidence assembled here, the structural class is at least as consequential for ideological hostility as any individual-level class, and its weaker evidence grade reflects the difficulty of randomizing institutions rather than a weaker expected effect. Mindfulness training and intergroup contact are separated because their evidence bases differ substantially. Figure 4 summarizes the mapping.

### 11.1. Targeting Valuation-Related Processing (OFC and vmPFC Nodes)

Education in epistemic pluralism and critical thinking is hypothesized to recalibrate how worth is assigned. Mastery-oriented and growth-mindset educational environments have been proposed as means of moderating maladaptive social comparison, and the findings are mixed. Burnette and colleagues [95] reported heterogeneous effects with stronger outcomes among at-risk subgroups. Macnamara and Burgoyne [96] reached more skeptical conclusions, attributing some observed effects to design and reporting heterogeneity. Growth-mindset interventions produce variable and modest effects, with stronger evidence in specific populations than in universal implementation. Integrating diverse belief systems into curricula remains theoretical at the valuation level, and its influence on out-group threat is hypothesized rather than demonstrated in controlled research.

### 11.2. Targeting Reward-Learning Signals (Ventral Striatum Node)

Platform redesign is the primary intervention aimed at reward reinforcement. Friction interventions, such as delays and confirmation prompts before sharing, are hypothesized to reduce the impulsive dissemination of emotionally charged content by interrupting rapid feedback loops. Pennycook and Rand [97] found that simple accuracy prompts reduced sharing of misinformation across platforms and populations, providing empirical support for the friction approach. Prosocial engagement metrics and civic recognition systems remain theoretical proposals with limited evidence connecting them to reduced ideological hostility. Most platform-level evidence derives from natural experiments and platform-internal A/B testing rather than independent randomized trials, and many findings are proprietary or incompletely reported.

### 11.3. Targeting Threat- and Salience-Related Processing (Salience-Network Nodes)

Two intervention classes are treated separately because their evidence bases differ. Mindfulness and emotion-regulation training have been linked to altered prefrontal regulation of amygdala reactivity in a substantial body of neuroscience literature reviewed by Tang, Holzel, and Posner [98]. Goyal and colleagues [99] meta-analyzed 47 randomized trials and reported small to moderate effects on anxiety, depression, and pain at eight weeks and at three to six months, with low or insufficient evidence for positive mood, attention, substance use, and sleep. Direct meta-analytic evidence that mindfulness training reduces ideological hostility or intergroup violence is absent, so effects on hostility are suggestive rather than demonstrated.

Intergroup contact under cooperative conditions has the strongest behavioral evidence base of any intervention considered here, and the most instructive dispute. Pettigrew and Tropp [100] meta-analyzed 713 independent samples from 515 studies and reported a significant inverse relationship between contact and prejudice, with larger effects under the conditions specified by Allport [101] and Sherif [102]. Paluck, Green, and Green [103] restricted the evidence base to studies with random assignment and delayed outcome measurement, found 27 such studies, and concluded that the policy-relevant evidence is narrower and more mixed than the aggregate meta-analytic estimate implies, with notable gaps including the absence of studies on interracial contact in adults over 25. The two results are compatible: contact is associated with reduced prejudice across a very large correlational and quasi-experimental literature, and the randomized evidence supporting contact as a deployable policy instrument is considerably thinner. Both are reported here, and the evidence grade in Table 3 reflects the design restriction rather than the aggregate count. The mega study conducted by Voelkel and colleagues [104], which tested 25 treatments in 32,059 participants, provides the strongest current randomized evidence on reducing partisan animosity and antidemocratic attitudes and found the largest effects for treatments highlighting sympathetic individuals with different political beliefs, for treatments emphasizing common identities, and, for antidemocratic attitudes specifically, for correcting misperceptions of rival partisans’ views. Perspective-taking has been proposed as a means of restoring mentalizing-related engagement toward stigmatized out-groups; Harris and Fiske [105] documented reduced engagement of mentalizing regions in response to extreme out-groups but did not test perspective-taking as an intervention, so the restorative hypothesis is a proposal aligned with an observed phenomenon rather than a result established by that work.

### 11.4. Structural and Socio-Ecological Recalibration of Status

Institutional fairness, transparent governance, and accessible grievance mechanisms are hypothesized to address the neuroendocrine component of the framework by reducing chronic status threat, and they are the intervention class most directly implied by Section 9.2 and Section 10.3. Civic honor frameworks that recognize inclusive achievement broaden the range of behavior treated as status-conferring. Three features justify this class receiving analytical parity with individual-level interventions despite its weaker evidence grade. It operates on the population rather than the individual and so does not require uptake by the people whose behavior is at issue, who are precisely those least likely to enroll in a mindfulness or contact program. It addresses the driver identified in Section 9.2 as social–ecological rather than neurochemical. And its failure mode is reversible through ordinary political means rather than through intervention in individuals’ lives. Its limitations are equally clear: it requires sustained political commitment, resists randomization, and relies predominantly on correlational and historical-comparative evidence. The structural class, therefore, carries the highest expected value and the lowest evidential resolution, which argues for investment in quasi-experimental designs exploiting institutional reform rather than for de-emphasis.

### 11.5. Evidence Grading Scheme for Table 3

The labels used in the intervention table in earlier versions of this work were not formally defined and were open to misinterpretation as expressing confidence in the neural mechanism. They are replaced by an explicit scheme, adapted from the GRADE logic [106] but restricted to what the present evidence supports, and applied to the behavioral outcome only. Grade A1 denotes multiple randomized trials with delayed outcome measurement plus independent meta-analytic synthesis. Grade A2 denotes meta-analytic synthesis of a large literature in which the randomized subset with delayed outcomes is limited or contested. Grade B1 denotes randomized or field-experimental evidence that is replicated but addresses a proximal rather than the target outcome. Grade B2 denotes randomized evidence with heterogeneous or contested effects. Grade C denotes correlational, historical–comparative, or purely theoretical support. Each grade describes the size and design of the behavioral evidence base for the intervention. No grade reflects the certainty of the proposed neural mechanism, which, in every case, remains hypothesized and correlational.

## 12. Design Guidelines for Preregistered Tests

### 12.1. Status of the Designs and Common Specification Requirements

Earlier versions of this work described the material below as preregistration-ready predictions while simultaneously stating that it was not preregistration-compliant. That was a contradiction, and it is resolved here in favor of the weaker and more accurate description. What follows are design guidelines for future preregistered studies. Each states a population, an intervention or design, a control, a primary outcome, and a falsification criterion, which is the information a preregistration must contain but is not itself a preregistration. Elements that remain at the discretion of an implementing team, principally the sample size and the associated power analysis, are identified as such rather than assumed.

Every preregistration built on these designs should specify in advance: the smallest effect size of interest, with Cohen’s d of 0.40 offered as a default for primary behavioral outcomes and a design-specific equivalent for imaging outcomes; target power of at least 0.80 with two-sided alpha of 0.05; the method of correction for multiple comparisons; inclusion and exclusion criteria together with procedures for motion artifact, attrition, and missing data; and the analysis plan, with any deviation reported transparently. Designs 1, 2, and 6 concern platform-level or longitudinal observational work in which sample size is determined by the available user base or by the smallest effect of interest. Designs 3, 4, 5, 7, 8, and 9 are neuroimaging designs and should meet contemporary power standards for social neuroscience, which, given the effect sizes reported in this literature, will generally require samples substantially larger than the 15 to 30 participants typical of the studies reviewed [55].

### 12.2. Imaging Specification Common to Designs 3, 4, 5, 7, 8, and 9

The following parameters are specified so that the imaging designs are operationally rather than nominally testable. Field strength: 3 T is the minimum, with 7 T permitted when the region of interest includes the amygdala or ventral striatum and susceptibility artifact can be controlled; results from different field strengths are not to be pooled. Primary analysis: multivariate pattern analysis is the primary method for all designs whose hypothesis concerns representational content, which includes Designs 4, 5, and 7, on the grounds set out in Section 4.4, where univariate overlap and multivariate separability gave opposite answers to the same question; univariate BOLD contrast is retained as the primary method only for Designs 3, 6, and 8, where the hypothesis concerns response magnitude within a predefined region rather than representational content. Primary contrast: each design below explicitly names its primary contrast, and no other contrast may be substituted after data collection. Activation versus connectivity: activation or pattern discriminability is the primary outcome in every design; functional connectivity is designated secondary and exploratory throughout, because connectivity analyses offer analytic flexibility that would compromise the falsification criterion if treated as primary. Region-of-interest definition: regions must be defined from independent anatomical atlases or from meta-analytic coordinates published before the study, never from the study’s own data.

### 12.3. The Designs

The guidelines for designs are summarized in Table 4.

## 13. Limitations and Ethical Considerations

### 13.1. Reverse Inference and Replication Constraints

As stated in Section 4.8, most of the neural evidence cited is correlational. Activation within a region does not establish exclusive functional specialization. Most cited neuroimaging research involves samples of 15 to 30 participants, is subject to publication bias favoring positive results, and uses paradigms that simplify the ecological complexity of real-world status dynamics. Replicability concerns in social neuroscience remain substantial [55]. The framework offers a plausible integrative organization of existing evidence and is not a confirmed causal architecture.

### 13.2. The Prestige Claim Is Narrower than the Title Suggests

The most serious limitation of this review is stated in Section 3.1 and repeated here because it qualifies every neural claim in Section 4, Section 5, Section 6, Section 7 and Section 8. The literature that supplies the framework’s neural content measures social rank, not prestige in the technical sense of freely conferred deference. The dominance–prestige distinction is behaviorally well supported but neurally untested. Consequently, the framework should be read as a candidate account of status processing in general, within which prestige is a hypothesized special case whose neural distinctiveness has not been demonstrated and is the subject of Design 9. Statements in this review that connect the neural literature to prestige specifically are extrapolations, are labeled as High inference in Table 1, and should not be cited as evidence of a neurobiology of prestige.

### 13.3. Cultural Generalizability

The neuroimaging and behavioral evidence base derive primarily from Western, educated, industrialized, rich, and democratic samples [107]. This limitation exceeds what the standard WEIRD caveat conveys. Cultural neuroscience documents variation in neural responses to self-referential, social, and value-relevant stimuli across cultural contexts, including differences in mPFC engagement during self-versus-other judgments and in reward and emotion processing as a function of cultural orientation [108]. Anthropological evidence documents substantial variation in what counts as prestige, sacred commitment, or status threat, and the cross-cultural heterogeneity in moral foundation weighting [77,78] gives that variation an empirical structure rather than leaving it as a general caution. Mapping a species-universal neural architecture onto culturally variable constructs risks assuming neural homogeneity, even though the evidence indicates cultural calibration, particularly at the algorithmic level. This framework holds that the large-scale networks and neuroendocrine output are broadly conserved across human populations while their calibration, the cues that engage them, and the meanings attributed to their states are co-determined by cultural and historical context (Section 4.9). Variation in the relative weight of dominance and prestige pathways, in cultural interpretation of status markers, in the content of sacred values, and in the distribution of identity fusion is not noise around a universal mechanism; it constitutes the constructs as they exist in populations. Cross-cultural neuroimaging and behavioral research that deliberately includes non-WEIRD samples and culturally calibrated stimuli is required before the policy implications of this framework can be generalized.

### 13.4. Risks and Boundaries of Mechanism-Aligned Intervention

Any proposal to redesign status incentives or modify digital reinforcement environments raises legitimate concerns about social engineering, paternalism, and the potential for authoritarian misuse. These risks require transparent governance, democratic oversight, and defined ethical boundaries. The framework favors environmental redesign over pharmacological or coercive modification of individual behavior, and the designs in Section 12 are limited accordingly. Environmental interventions are not ethically neutral, and the neutrality of any intervention class should be assessed alongside its mechanistic plausibility.

### 13.5. Alternative Explanatory Frameworks and the Question of Marginal Utility

The proposed framework is not the only account capable of explaining status-related phenomena, and a new framework earns its place only if it generates predictions that established alternatives cannot. This subsection assesses four alternatives against that standard rather than listing them.

Economic rational-choice models explain status competition through utility maximization and require no neural-level explanation, accounting for escalation under scarcity, positional goods, and costly signaling with fewer assumptions than the present framework. They do not predict domain-general habituation of the response to status feedback, since habituation is a property of a learning process rather than of a preference ordering, nor that identity-relevant and non-identity-relevant rewards should dissociate in their resistance to devaluation. Structural inequality models explain status-related distress through material power relations and institutional arrangements, account well for the association between institutional legitimacy and population-level outcomes, and reach the policy recommendation of Section 11.4 by a shorter route. They do not predict that the same institutional condition should produce different physiological profiles as a function of individual threat appraisal, nor specify why some grievances become non-negotiable while others of equal material severity do not.

Social identity theory [16] explains intergroup dynamics through categorization and self-esteem maintenance, and is the most direct competitor, predicting in-group favoritism, out-group derogation under threat, and the effects of cross-cutting categorization, as attributed in Section 10.4 to identity complexity. It does not predict the asymmetry central to Section 7, namely that commitments processed as sacred resist compromise in a manner qualitatively rather than quantitatively different from strongly held preferences, nor that willingness to sacrifice should be associated with reduced rather than increased deliberative processing. Dual-process moral psychology, including the fMRI evidence of Greene and colleagues [109], explains resistance to utilitarian trade-offs through competition between intuitive and deliberative processes and accounts for taboo trade-off aversion without a status framework. It does not predict that sacred-value processing should recruit rule-retrieval regions in the manner reported by Berns and colleagues [22], and it treats deliberative processing as opposing rather than as disengaged, which is the opposite of the pattern reported in radicalized samples [19,20]. Moral foundations theory [77,78] is a fifth alternative with respect to Section 7, and, as noted in Section 7.3, it predicts that the present framework cannot, namely, that the content of a violated commitment shapes the response independently of its sacredness.

Table 5 states which of the designs in Section 12 discriminate against the present framework. The honest summary is that the framework’s marginal utility rests on a small number of discriminating predictions, principally the convergence of different sacred-value contents on a common processing signature (Design 4), the domain-general habituation of status-feedback responses (Design 6), and the neural separability of prestige from dominance-derived rank (Design 9). If those three fail, the framework should be regarded as an organizing vocabulary rather than an explanatory advance over the alternatives above, and this review should state that criterion in advance rather than after the results are known.

## 14. Conclusions: Redirecting the Status Brain

Human pride is neither pathology nor virtue. It is the experiential signature of neural processing that contributes to the regulation of social status, shaped by evolutionary pressures favoring the detection of hierarchy, social learning, and cooperative coordination. The framework proposed here groups five categories of functionally pluripotent processing, namely valuation-related, reward-learning, self-referential and identity-relevant, threat- and salience-related, and neuroendocrine output, and treats them as engagement of nodes within overlapping large-scale networks rather than as five dissociable mechanisms, with a candidate computational signal now specified for each. It treats material ownership, religious and ideological belief, digital prestige, and self-sacrifice as candidate domains of application. The strongest direct neural evidence concerns social rank and material ownership; sacred-value processing and adolescent peer feedback are intermediate; digital prestige systems and ideological hostility rest on limited or extrapolated evidence. Prestige in the strict sense of freely conferred deference has not been isolated neurally at all, and the framework is correspondingly a framework for status processing within which prestige remains a hypothesis.

Modern environments may amplify status-relevant processing in ways that diverge from the conditions under which these processes evolved. Consumer markets exploit scarcity signaling and authenticity narratives. Sacred-value absolutism resists compromise when fused with identity and political power. Digital platforms generate variable and intermittent social feedback capable of sustaining motivational engagement, with platform-level polarization effects that vary across regimes and outcomes [81] and include experimental evidence of cross-cutting exposure backfiring in some populations [37]. The mismatch between ancestral neural architecture and contemporary symbolic environments is a plausible account of several modern social phenomena; the causal relationship at the neural level remains hypothetical.

When the problem is ideological hostility rather than clinical aggression, the evidence favors institutional and environmental redesign over pharmacological suppression. Future research should test whether reallocating status incentives, strengthening regulatory capacity through educational and contemplative practice, broadening identity complexity, designing digital environments that reward deliberation, and building institutions that reduce chronic humiliation produce measurable reductions in intergroup hostility. Each map to a component of the framework, with evidence strength graded in Section 11.5, and the structural class carries the highest expected value alongside the weakest evidential resolution, which argues for better designs rather than lower priority.

A society without conflict is biologically improbable given the depth of the evolutionary origins of status competition and in-group favoritism. Historical evidence nevertheless indicates that societies can substantially reduce discrimination, hostility, and violence by building institutions that work with rather than against evolved psychological mechanisms. Some large-scale neural substrates may be broadly conserved across human populations, while their calibration, the cues that engage them, and the social meanings of the resulting states vary across cultural and historical contexts. The high-level constructs of prestige, status, and sacred value emerge from these interactions rather than being fixed properties of neural tissue (Section 4.7, Section 4.9 and Section 13.3). Understanding this architecture does not eliminate risk, but it can inform deliberate design toward prosocial stability.

## Figures and Tables

**Table 2:** Candidate computational signal, model class, and estimable parameter for each processing category. None of these mappings is established specifically for status contexts; each is a hypothesis that renders the corresponding design in Section 12 parametric rather than directional.

Processing Category	Candidate Computational Signal	Model Class	Estimable Parameter and Status-Relevant Claim	Key Refs
Valuation-related processing (OFC, vmPFC)	Comparison of subjective values on a common scale	Value-based decision models in which choice probability is a function of value difference	Value-difference sensitivity; cultural learning enters as a modifier of the value function rather than as a separate signal, which is the computational statement of the labeling effects in Section 4.1	[5,24]
Reward-learning signals (ventral striatum)	Reward prediction error	Temporal-difference reinforcement learning	Learning rate and prediction-error weight fitted to social and non-social feedback in the same participants; striatal prediction-error responses appear domain-general rather than socially specialized	[40,42]
Self-referential and identity-relevant processing (mPFC, DMN)	Bayesian belief updating over self-relevant propositions, including own position in a hierarchy	Bayesian inference over latent power or standing	Prior precision and update magnitude; a Bayesian scheme tracking individuals’ power fits behavior and neural data better than a chess-style rating model, and mPFC selectively mediates updating about one’s own hierarchy	[9]
Threat- and salience-related processing (salience network)	Precision weighting, that is, allocation of learning rate and attentional gain by estimated reliability and relevance	Predictive processing and active inference	Precision parameter; status threat predicted to raise learning rate for identity-relevant evidence and lower it for identity-inconsistent evidence, a parametric prediction unavailable from a regional description	[52]
Neuroendocrine output (HPA axis)	Slow gain control over the preceding signals rather than a signal in its own right	Neuromodulatory gain and arousal models	Gain or temperature parameter; cortisol elevation predicted to shift the balance between model-based and habitual control and to compress the range over which precision is adjusted	[11,12,48]

Two further model classes bear on the collective phenomena of Section 7 and Section 8. multi-agent reinforcement learning formalizes how cooperation and conflict emerge from individually rational learning in sequential social dilemmas [53] and is the formalism in which the claim of Section 8 that platform incentives reshape expression becomes stimulable rather than narratable. Computational accounts of mental-state attribution formalize mentalizing as rational inference over others’ beliefs, desires, and percepts [54], which is the level at which the perspective-taking interventions of Section 11.3 should be specified. Practical constraints on fitting such models to neuroimaging data are reviewed by Lockwood and Klein-Flugge [42].

**Table 3:** Candidate intervention classes mapped to processing and network targets, with evidence grades assigned under the scheme defined in Section 11.5. Mindfulness and intergroup contact are listed separately because their evidence bases differ.

Intervention	Hypothesized Processing Target	Evidence Grade (Behavioral Outcome Only)	Scalability	Risks and Limitations
Epistemic pluralism/critical-thinking education	Valuation-related processing engaging OFC and vmPFC nodes	B2: randomized evidence with heterogeneous and contested effects [95,96]	Moderate	Effect heterogeneity; weak universal effects
Platform friction/accuracy prompts	Reward-learning signals indexed in ventral striatum	B1: replicated field-experimental evidence on a proximal outcome, namely misinformation sharing rather than hostility [97]	Very high	Industry resistance; user attrition; proprietary platform evidence
Identity-complexity education	Self- and identity-relevant processing engaging mPFC within default-mode network	B2: theoretical base established [94]; intervention trials sparse	High	Long-term effects understudied; few intervention trials
Mindfulness/emotion regulation	Threat- and salience-related processing involving salience-network nodes	B1: meta-analysis of 47 randomized trials on psychological stress outcomes; no direct evidence on ideological hostility [98,99]	Moderate	Compliance varies; cultural acceptability varies; target outcome not measured
Cooperative intergroup contact	Threat- and salience-related processing involving salience-network nodes	A2: large meta-analytic literature [100,101,102]; randomized subset with delayed outcomes is limited and contested [103]; strongest randomized evidence from a 25-treatment megastudy [104]	Moderate	Poorly designed contact may backfire [37]; randomized policy evidence thinner than aggregate estimate implies
Institutional fairness/rule of law	Neuroendocrine output and slow modulation of the preceding categories	C: correlational and historical–comparative	Population-level	Slow; requires political commitment; resists randomization
Civic honor/public recognition	Reward-learning signals indexed in ventral striatum	C: theoretical	Moderate to high	Definition of prosocial is contested

Evidence grades follow Section 11.5 and describe the size and design of the behavioral evidence base for each intervention. They do not express confidence in the proposed neural mechanism, which is hypothesized and correlational in every row. The processing targets in column two are candidate targets inferred from the framework, not demonstrated mechanisms of action.

**Table 4:** Design guidelines for preregistered tests. Each row states the information a preregistration must contain and is not itself a preregistration; sample size and the associated power analysis remain with the implementing team.

Design	Population	Intervention or Design, and Control	Primary Outcome and Contrast	Falsification Criterion	Confounds and Secondary Measures
1. Reward recalibration	Users of one or more major social media platforms	Redirection of engagement metrics toward prosocial content categories, or an analogous civic recognition campaign; control is a matched cohort or pre-intervention period	Composite engagement-shift index: proportion of high-engagement posts classified as cooperative rather than outrage-laden	No detectable shift over the pre-specified window, or a shift in the opposite direction	Secular trends, content-classifier drift, concurrent algorithmic change
2. Platform friction	Active users of a platform supporting A/B testing	Deliberation prompts, comprising a sharing delay and a confirmation step, before posting emotionally charged content; control is the standard sharing procedure	Rate of sharing emotionally charged content per active user, using a pre-specified classifier	No reduction in the primary outcome at the pre-specified power	Novelty effects, classifier drift
3. Identity-complexity training	High-school or university students with no prior identity-complexity intervention	Structured curriculum emphasizing multiple cross-cutting identities; control is an equivalent-duration social studies curriculum	Univariate amygdala BOLD response in an anatomically defined ROI, pre and post. Contrast: ideologically challenging minus neutral	No pre-post change in the amygdala ROI relative to control, or change in the opposite direction	Connectivity is secondary and exploratory only
4. Sacred-value priming (tiered; see note)	Tier 1: population matched to the source samples on ideological intensity, using a validated intensity measure as an inclusion criterion. Tier 2: adults with sacred values across at least two moral foundations but without indicators of extremity	Sacred-value priming before a willingness-to-compromise task; control is neutral non-sacred priming	Multivariate discriminability of sacred from non-sacred compromise decisions in dlPFC, IFG, and parietal cortex. Contrast: sacred minus non-sacred compromise	Classifier at chance in all three pre-specified regions, or a pattern inconsistent with reduced cost–benefit deliberation	vmPFC to dlPFC connectivity is secondary and exploratory
5. Contact and mentalizing	Members of two groups with a documented history of intergroup tension	Structured cooperative contact under Allport-style conditions of equal status, shared goals, and institutional support; control is a parallel-duration non-contact program	Multivariate separability of out-group from in-group exemplars in mentalizing regions defined from prior meta-analytic coordinates, pre and post; prediction is reduced separability. Contrast: out-group minus in-group	No pre-post change in separability relative to control, or change confined to in-group exemplars	Delayed behavioral prejudice measures to be reported regardless of imaging result, given the design gap identified in [103]
6. Digital habituation	Frequent users of social media platforms	Longitudinal observational design over a minimum of six months; no intervention	Univariate ventral striatal BOLD response to standardized like-feedback across time points. Contrast: high minus low like count	No longitudinal attenuation, or a pattern inconsistent with prediction-error habituation	Fitted learning-rate parameters are secondary and would convert this to a parametric test
7. Pride facets and contemplative practice	Adults beginning long-term compassion-focused meditation, stratified at baseline by authentic and hubristic pride scores	Meditation program; control is a matched waitlist	Multivariate separability of self from other trials in mPFC (predicted to decrease); of compassion from neutral trials in anterior insula and ACC (predicted to increase); and self-reported hubristic pride and status sensitivity (predicted to decrease) paired with authentic pride and prosocial motivation (stable or increased)	No change in any of the three pre-specified primary outcomes after the pre-specified training duration	Also a partial test of the conjectured pride separation in Section 3.3
8. Institutional legitimacy	Matched samples from countries with high and low institutional trust indices	Observational cross-sectional comparison, with covariate adjustment for individual socioeconomic position	Baseline morning cortisol and amygdala reactivity to status-threatening stimuli in a standardized task	No association between institutional trust and either the neural or the endocrine outcome after adjustment	A quasi-experimental version exploiting a documented institutional reform with pre-reform and post-reform measurement is preferable where available
9. Prestige versus rank (see note)	Adults with no prior relationship to the target individuals	Within-participants comparison of two hierarchy-inducing conditions matched on rank information but differing in source: rank by contest outcome (dominance) versus rank by freely conferred third-party deference following demonstrated competence (prestige)	Multivariate discriminability of prestige-derived from dominance-derived rank in mPFC, amygdala, and ventral striatum. Contrast: prestige minus dominance at matched rank distance	Classifier at chance in all pre-specified regions, which would indicate that the reviewed neural evidence indexes rank generally and licenses no prestige-specific claim	Logically prior to Designs 1 to 8; see Section 3.1 and Section 13.2

Note on Design 4. The population restriction is a substantive improvement relative to earlier versions of this work. The neural findings on which the design rests [19,20] were obtained in samples selected for ideological extremity, and extending a prediction derived from them to adults with sacred values in general would commit the generalization this review warns against in Section 3.4. Tier 1 is therefore the confirmatory test and Tier 2 an explicit generalization test whose failure would bound the population of the mechanism rather than falsify it. An effect present in Tier 1 and absent in Tier 2 would indicate a mechanism of ideological intensity rather than of sacredness as such, substantially narrowing the claims in Section 7. Note on Design 9. Until this design or an equivalent is conducted, every prestige-specific neural statement in this literature, including those in this review, remains an extrapolation from rank paradigms.

**Table 5:** Discriminating predictions. For each design in Section 12, the table indicates whether the same prediction can be derived from each alternative framework. A prediction that every alternative also generates does not contribute to the marginal utility of the present framework.

Design (Section 12.3)	Rational Choice	Structural Inequality	Social Identity Theory	Dual-Process Moral Psychology	Discriminating?
1. Reward recalibration	Yes	Partly	Yes	No	No
2. Platform friction	Yes	No	Partly	Yes	No
3. Identity-complexity training	No	No	Yes	No	No
4. Sacred-value priming, tiered	No	No	No	Partly, but predicts increased rather than reduced deliberative engagement	Yes
5. Contact and mentalizing	No	Partly	Yes	No	No
6. Digital habituation	No, preference orderings do not habituate	No	No	No	Yes
7. Pride facets and contemplative practice	No	No	Partly	No	Partly
8. Institutional legitimacy	Partly	Yes	Partly	No	No
9. Prestige versus rank	No	No	Partly	No	Yes

Partly indicates that the alternative predicts the direction of the effect but not the specific outcome measure or the mechanism specified in Section 12.3. The three designs marked Yes constitute the framework’s testable claim to explanatory value beyond established alternatives.

## Data Availability

No new data were created or analyzed in this study. Data sharing is not applicable to this article.

## Supplementary Materials

The following supporting information can be downloaded at: https://www.mdpi.com/article/10.3390/brainsci16080847/s1, Table S1, study-level characteristics of all studies supporting a neural or behavioral claim in Section 3, Section 4, Section 5, Section 6, Section 7, Section 8, Section 9, Section 10 and Section 11, reporting sample size, paradigm, replication status, and principal limitation.
