# Supplementary material for: Prestige, Pride, and Belief: A Hypothesized Neural Integration Framework for Status, Identity, and Modern Polarization
Source: Brain Sci. 2026 Aug 10;16(8):847. doi: 10.3390/brainsci16080847 (PMC13510474; doi:10.3390/brainsci16080847)
Supplement: Supplementary file 1 [file brainsci-16-00847-s001.zip › brainsci-4330040-supplementary.pdf]

Supplementary Materials

Prestige, Pride, and Belief: A Hypothesized Neural Integration Framework for Status, Identity, and Modern Polarization

Sarfaraz K. Niazi, College of Pharmacy and Pharmaceutical Sciences, Washington State University, Spokane, WA 99202, USA

Table S1. Study-level characteristics of the evidence base

This table lists every study that supports a neural, behavioral, or intervention claim in Sections 3 through 11 of the main text. It is provided so that readers can audit the selection underlying a selective narrative review rather than accept it. Studies are ordered by reference number. The Evidence type column uses the three-way classification defined in Section 2.3 of the main text. The Replication column records whether the specific finding cited has been independently replicated, meta-analytically synthesized, or subjected to a documented replication failure; it does not describe the general standing of the research program. Entries marked with an asterisk in the Sample column report values verified against the source during revision; for the remaining primary fMRI studies the participant numbers fall in the range of approximately 15 to 40 that is characteristic of this literature and that Section 4.8 and Section 13.1 identify as a structural limitation of the evidence base, and the exact figures should be read from the cited sources.

Table S1. Studies supporting neural, behavioral, and intervention claims in Sections 3 to 11.

Ref	Study	Paradigm or design	Sample	Replication status	Principal limitation for present use
[1]	Henrich & Gil-White 2001	Theoretical synthesis of prestige as social-learning adaptation	Not applicable	Widely adopted framework; not an empirical test	Theoretical; supplies the construct, not neural evidence
[2]	Cheng, Tracy & Henrich 2013	Behavioral and peer-rating studies distinguishing dominance and prestige routes to rank	Multi-study behavioral	Construct distinction replicated behaviorally across labs	Behavioral only; no neural operationalization of prestige
[3]	Tracy & Robins 2007	Factor-analytic studies of pride structure	Multi-sample psychometric	Two-facet structure replicated psychometrically	Psychometric; no neural data
[4]	Rolls 2000	Review of OFC reward coding, primate and human	Review	Core findings well established	Review; not a status paradigm
[5]	Rangel, Camerer & Montague 2008	Theoretical framework for value-based decision making	Review	Framework standard in neuroeconomics	Framework, not evidence; non-social valuation
[6]	Northoff et al. 2006	Meta-analysis of self-referential processing imaging studies	Meta-analysis of imaging studies	Meta-analytic	Self-referential processing is not identity integration; reverse-inference constraint applies
[7]	Sapolsky 2005	Observational primate hierarchy and glucocorticoid work	Non-human primates	Extensively replicated in primates	Non-human; concerns hierarchy generally, not human prestige
[8]	Tracy, Shariff & Cheng 2010	Theoretical review of pride facets	Review	Not an empirical test	Review; supports construct only
[9]	Tajfel & Turner 1979	Social identity theory	Theoretical	Foundational; extensively tested	Theory, treated in main text as a competing framework
[10]	Swann et al. 2009	Identity fusion scales and willingness-to-sacrifice measures	Multi-study behavioral	Behaviorally replicated across samples and countries	Behavioral only; no neural measure of fusion
[11]	Cikara, Botvinick & Fiske 2011	fMRI of intergroup competition and harm (sports rivalry)	Primary fMRI	Not independently replicated at scale	Concerns competition and harm, not identity fusion; small sample
[12]	Hamid et al. 2019	fMRI of willingness to fight and die for sacred vs non-sacred values	Primary fMRI; supporters of an Al Qaeda associate	Partially converging with [13]; no independent replication	Population selected for ideological extremity; not generalizable (Section 3.4; Design 4)
[13]	Pretus et al. 2018	fMRI of sacred values and vulnerability to violent extremism	Primary fMRI; Spanish sample with sacralized values	Partially converging with [12]	Same population restriction as [12]
[14]	Atran & Ginges 2012	Review and field studies of sacred values in conflict	Review and field data	Field findings replicated across conflict settings	Not neural

Ref	Study	Paradigm or design	Sample	Replication status	Principal limitation for present use
[15]	Berns et al. 2012	fMRI of personal sacred vs non-sacred statements	Primary fMRI	Not independently replicated	Small sample; TPJ and vlPFC interpretation subject to reverse inference
[16]	Lynn 1991	Quantitative review of commodity theory and scarcity effects	Quantitative review	Superseded in part by later valuation work	Dated (1991); updated in main text with [106]
[17]	Zink et al. 2008	fMRI of stable and unstable social hierarchy viewing and competition	Primary fMRI	Converging with later rank-learning work [97,98]	Operationalizes rank, not prestige (Section 3.1)
[18]	Plassmann et al. 2008	fMRI of price labeling and experienced pleasantness of wine	Primary fMRI	Conceptually replicated in marketing neuroscience; effect size varies	Small sample; single stimulus class
[19]	Kim & Johnson 2012	fMRI of transient self-object association	Primary fMRI	Converging with ownership literature	Small sample; ownership manipulation is transient and laboratory-based
[20]	Kirk et al. 2009	fMRI of semantic context (gallery label) and aesthetic value	Primary fMRI	Not independently replicated	Small sample; aesthetic domain only
[21]	McClure et al. 2004	fMRI of brand labeling and preference for familiar drinks	Primary fMRI	Widely cited; direct replications limited	Small sample; single product category
[22]	Sherman et al. 2016	fMRI of like-count feedback in a simulated Instagram task	32 adolescents*	Not replicated at scale; no adult or platform-level replication	Single study; adolescent; simulated environment. Carries the entire direct neural claim in Section 8 (Figure 3, link a)
[23]	Crockett 2017	Theoretical account of moral outrage in digital environments	Review	Not an empirical test	Theoretical; frequently miscited as evidence
[24]	Montag et al. 2019	Theoretical and integrative discussion of addictive platform features	Review	Not an empirical test	Explicitly not a demonstration of platform-level reinforcement schedules
[25]	Izuma, Saito & Sadato 2008	fMRI comparing social and monetary reward in the striatum	Primary fMRI	Converging with domain-general prediction-error findings [99]	Small sample; social reward operationalized as approving faces
[26]	Eisenberger & Lieberman 2004	Review proposing a common alarm system for physical and social pain	Review	Strong reading contradicted by [90,91]	Predates multivariate methods; cited in main text together with its refutation (Section 4.4)
[27]	Brady et al. 2021	Observational and experimental study of social learning and outrage expression	Large-scale online behavioral	Converging with [28,29]	Behavioral only; no neural measure
[28]	Brady et al. 2017	Analysis of moral-emotional language and diffusion in a large Twitter corpus	Large social media corpus	Converging with [27,29]; effect size debated	Correlational; no neural measure; within-network effect larger than between-network
[29]	Rathje, Van Bavel & van der Linden 2021	Multi-platform analysis of out-group animosity and engagement	Large multi-platform corpora	Converging with [27,28]	Correlational; engagement is a platform metric, not hostility
[30]	Bail et al. 2018	Field experiment: paid partisans followed opposing-ideology bots	Randomized field experiment, U.S. partisans	Independent replications limited	Effect significant among Republicans only; trend non-significant among Democrats
[31]	Schultz 1998	Primate electrophysiology of dopamine prediction-error signaling	Non-human primate	Extensively replicated	Non-human; non-social reward
[32]	Berridge & Robinson 2003	Review distinguishing liking and wanting	Review	Framework well supported	Review; not a social or status paradigm
[33]	Wu et al. 2017	Competition manipulation, conspicuous consumption, and testosterone	Behavioral with endocrine measures	Endocrine prediction not confirmed	Reported here as a null result for testosterone reactivity; cited as disconfirming evidence
[35]	Tamir & Mitchell 2012	fMRI and behavioral studies of self-disclosure as intrinsically rewarding	Primary fMRI plus behavioral	Behavioral component replicated	Laboratory disclosure; not a platform setting
[36]	Kaplan, Gimbel & Harris 2016	fMRI of counterevidence against political beliefs	40 self-identified U.S. liberal participants*	Not independently replicated	Single ideological group in one country; cannot support general claims about belief
[37]	Botvinick, Cohen & Carter 2004	Review of conflict monitoring and ACC	Review	Core account well supported, later contested in detail	Review; non-social conflict paradigms

Ref	Study	Paradigm or design	Sample	Replication status	Principal limitation for present use
[38]	Dickerson & Kemeny 2004	Meta-analysis of acute stressors and cortisol response	Meta-analysis	Meta-analytic	Laboratory stressors; social-evaluative threat operationalized narrowly
[39]	Poldrack 2006	Methodological analysis of reverse inference	Methodological	Standard reference	Methodological constraint, not evidence
[40]	Button et al. 2013	Analysis of statistical power across neuroscience	Meta-research	Widely confirmed	Applies to nearly every primary fMRI study in this table
[41]	Thomsen et al. 2011	Infant looking-time study of dominance representation	Infants aged 8 to 13 months	Replication record mixed in infant cognition generally	Looking-time inference; developmental generalization is indirect
[42]	Chudek et al. 2012	Preschool learning from knowledgeable vs dominant models	Preschool children	Converging with prestige-bias literature	Behavioral; small developmental samples
[45]	Gelfand et al. 2011	Survey study of cultural tightness and looseness	33 nations*	Broadly replicated with debate on measurement	Nation-level survey; ecological inference
[54]	Kapogiannis et al. 2009	fMRI of religious belief statements	Primary fMRI	Not independently replicated	Small sample; predominantly Western Christian participants
[56]	Inzlicht & Tullett 2010	ERP study of religious priming and error-related negativity	Primary EEG	No adequately powered independent replication established	Small-sample ERP; effect reported in main text as suggestive only (Section 7.1)
[57]	Granqvist et al. 2010	Review of religion as attachment	Review	Psychological construct supported; neural claim untested	Neural overlap with parental bonding circuitry is a hypothesis, not a finding
[60]	Lorenz-Spreen et al. 2023	Systematic review of digital media and democracy	Systematic review, worldwide evidence	Systematic	Effects mixed and regime-dependent; cited as disconfirming a uniform polarization claim
[61]	Jones et al. 2011	Meta-analysis of mood stabilizers for impulsive or repetitive aggression	10 placebo-controlled trials*	Meta-analytic	Pooled effect non-significant when restricted to low risk-of-bias studies
[63]	van Schalkwyk et al. 2018	Meta-analysis of antipsychotics for aggression in adults	21 trials*	Meta-analytic	SMD approximately 0.29; no clear superiority among agents
[65]	VA/DoD 2023	Clinical practice guideline, version 4.0	Guideline	Guideline consensus	Pharmacotherapy targets PTSD symptoms, not hostility as such
[69]	Border et al. 2019	Large-sample tests of historical candidate-gene hypotheses	Multiple large samples	Definitive non-replication	Cited as disconfirming evidence against candidate-gene moderators
[73]	Roccas & Brewer 2002	Social identity complexity framework and measures	Theoretical plus measurement	Measure adopted widely; intervention trials sparse	Few intervention trials; basis for a B2 grade in Table 3
[74]	Burnette et al. 2023	Systematic review and meta-analysis of growth-mindset interventions	Meta-analysis	Meta-analytic	Heterogeneous effects; stronger in at-risk subgroups only
[75]	Macnamara & Burgoyne 2023	Systematic review and meta-analysis of growth-mindset interventions	Meta-analysis	Meta-analytic; reaches more skeptical conclusion than [74]	Cited alongside [74] as an unresolved disagreement
[76]	Pennycook & Rand 2022	Accuracy-prompt experiments across platforms and populations	Multiple field and online experiments	Replicated and generalized by the authors	Outcome is misinformation sharing, not hostility; proximal outcome only
[77]	Tang, Holzel & Posner 2015	Review of mindfulness meditation neuroscience	Review	Review of a heterogeneous literature	Neural claims correlational; hostility not an outcome
[78]	Goyal et al. 2014	Meta-analysis of meditation programs	47 randomized trials*	Meta-analytic	Small to moderate effects on anxiety, depression, pain; low or insufficient evidence for other outcomes
[79]	Pettigrew & Tropp 2006	Meta-analysis of intergroup contact and prejudice	713 independent samples from 515 studies*	Meta-analytic	Predominantly correlational and quasi-experimental; see [107]
[82]	Harris & Fiske 2006	fMRI of responses to extreme out-groups	Primary fMRI	Partially replicated; interpretation contested	Documents a phenomenon; does not test perspective-taking as an intervention
[83]	Henrich, Heine & Norenzayan 2010	Comparative review of sample composition in behavioral science	Review with comparative database	Extensively confirmed	Applies to nearly every primary study in this table
[84]	Greene et al. 2001	fMRI of emotional engagement in moral judgment	Primary fMRI	Widely replicated; dual-process interpretation contested	Cited in main text as a competing framework

Ref	Study	Paradigm or design	Sample	Replication status	Principal limitation for present use
[89]	Han & Northoff 2008	Review of transcultural neuroimaging	Review	Review	Supports cultural calibration claim; limited non-WEIRD primary data
[90]	Wager et al. 2013	Multivariate fMRI signature of physical pain	Primary fMRI, multiple studies	Signature validated in independent samples	Cited as constraining the social-pain overlap claim
[91]	Woo et al. 2014	Multivariate discrimination of physical pain and social rejection	60 participants, plus 91 in an independent resting-state dataset*	Method and finding independently supported	Cited as disconfirming the functional-continuity reading of [26]
[92]	Mehta & Josephs 2010	Dual-hormone hypothesis: testosterone by cortisol interaction on dominance	Two studies; Study 2 n = 57 with complete hormone data*	Meta-analytically supported but weakly [93]	Interaction small and heterogeneous; stronger in males and for explicit status indices
[93]	Dekkers et al. 2019	Meta-analysis of the dual-hormone hypothesis	33 studies, 49 effect sizes*	Meta-analytic	Effect small with substantial heterogeneity in direction and magnitude
[94]	De Dreu et al. 2010	Double-blind placebo-controlled intranasal oxytocin, intergroup economic games	Three experiments, male participants	Tend-and-defend pattern partially replicated; field contested	Male-only; laboratory economic games; wider oxytocin literature has weak replication record [96]
[95]	Ditzen et al. 2009	Double-blind placebo-controlled intranasal oxytocin during couple conflict	Randomized crossover, couples	Not independently replicated at scale	Dyadic conflict, not intergroup; small sample
[96]	Declerck et al. 2020	Registered replication of oxytocin and trust	High-powered registered report (power > 95%)*	Definitive non-replication of the original finding	Cited as disconfirming evidence; constrains all oxytocin claims in Section 4.5
[97]	Kumaran et al. 2016	Model-based fMRI of social hierarchy learning; Bayesian vs reinforcement-learning models	Primary model-based fMRI	Converging with [98]	Operationalizes rank, not prestige; laboratory hierarchy
[98]	Qu et al. 2017	Interdisciplinary review of social dominance hierarchies	Review	Review	Concerns dominance and rank; does not address freely conferred deference
[99]	Lockwood & Klein-Flugge 2021	Methodological primer on reinforcement-learning models in social neuroscience	Methodological review	Standard reference	Methodological; also the source for the domain-generality caveat on striatal prediction error
[100]	Baker et al. 2017	Computational model of quantitative mental-state attribution	Behavioral model fitting	Model class independently developed	Not a status paradigm; cited as the level at which mentalizing should be specified
[101]	Leibo et al. 2017	Multi-agent reinforcement learning in sequential social dilemmas	Simulation	Simulation results reproduced in subsequent work	Simulation, not human data; cited as a formalism, not as evidence
[102]	Friston 2010	Free-energy principle and active inference	Theoretical	Framework widely adopted and widely contested	Theoretical; cited as the alternative decomposition that could supersede the five-category taxonomy
[103]	Haidt & Graham 2007	Moral foundations theory: theoretical and empirical case for five foundations	Theoretical with survey evidence	Foundational; measurement debated	Cited as a competing and complementary framework (Sections 7.3 and 13.5)
[104]	Graham et al. 2013	Review and empirical evaluation of moral foundations theory	Review of a large empirical program	Extensively tested; number of foundations contested	Supplies cross-cultural heterogeneity in moral content
[105]	Schjoedt et al. 2009	fMRI of improvised vs formalized personal prayer	Primary fMRI; Danish Christian participants	Not independently replicated	Small, culturally narrow, single-tradition sample
[106]	Bartra, McGuire & Kable 2013	Coordinate-based meta-analysis of subjective value	Meta-analysis of BOLD fMRI experiments	Meta-analytic	Non-social valuation predominates; used to update [16]
[107]	Paluck, Green & Green 2019	Re-evaluation of contact hypothesis restricted to randomized designs with delayed outcomes	27 qualifying studies*	Systematic	No study assesses interracial contact in adults over 25; basis for the A2 rather than A1 grade in Table 3
[108]	Voelkel et al. 2024	Megastudy of 25 crowdsourced treatments for partisan animosity and antidemocratic attitudes	32,059 participants*	Single very large randomized study; replication pending	U.S. only; outcomes are attitudinal and short-term
[109]	Guyatt et al. 2008	GRADE approach to rating evidence quality	Methodological	Standard	Adapted rather than applied in full; see Section 11.5

Asterisk denotes a sample or study count verified against the source during revision. Studies cited in the main text solely for theoretical framing, historical attribution, or definitional purposes and not supporting an empirical claim are omitted from this table. Where a study appears in the main text as disconfirming evidence, the Principal limitation column records that role explicitly, since these entries are the audit trail for the bias-mitigation procedure described in Section 2.5.
